# Assessment of the course of acute pancreatitis in the light of aetiology: a systematic review and meta-analysis

**DOI:** 10.1038/s41598-020-74943-8

**Published:** 2020-10-21

**Authors:** Emese Réka Bálint, Gabriella Fűr, Lóránd Kiss, Dávid István Németh, Alexandra Soós, Péter Hegyi, Zsolt Szakács, Benedek Tinusz, Péter Varjú, Áron Vincze, Bálint Erőss, József Czimmer, Zoltán Szepes, Gábor Varga, Zoltán Rakonczay

**Affiliations:** 1 Department of Pathophysiology, University of Szeged, Szeged, Hungary; 2 Institute for Translational Medicine and Szentágothai Research Centre, University of Pécs, Pécs, Hungary; 3 MTA-SZTE Momentum Translational Gastroenterology Research Group, Szeged, Hungary; 4 Clinical Medicine Doctoral School, University of Szeged, Szeged, Hungary; 5 First Department of Medicine, University of Pécs, Pécs, Hungary; 6 First Department of Medicine, University of Szeged, Szeged, Hungary; 7 Department of Oral Biology, Semmelweis University, Budapest, Hungary

**Keywords:** Gastrointestinal diseases, Pancreas, Gastrointestinal diseases

## Abstract

The main causes of acute pancreatitis (AP) are biliary disease, alcohol consumption, hypertriglyceridaemia (HTG) and endoscopic retrograde cholangiopancreatography (ERCP). The aim of this meta-analysis was to evaluate the effects of these aetiological factors on the severity and outcome of AP. Pubmed and Embase were searched between 01/01/2012 and 31/05/2020. Included articles involved adult alcoholic, biliary, HTG- or post-ERCP AP (PAP) patients. Primary outcome was severity, secondary outcomes were organ failures, intensive care unit admission, recurrence rate, pancreatic necrosis, mortality, length of hospital stay, pseudocyst, fluid collection and systematic inflammatory response syndrome. Data were analysed from 127 eligible studies. The risk for non-mild (moderately severe and severe) condition was the highest in HTG-induced AP (HTG-AP) followed by alcoholic AP (AAP), biliary AP (BAP) and PAP. Recurrence rate was significantly lower among BAP vs. HTG-AP or AAP patients (OR = 2.69 and 2.98, 95% CI 1.55–4.65 and 2.22–4.01, respectively). Mortality rate was significantly greater in HTG-AP vs. AAP or BAP (OR = 1.72 and 1.50, 95% CI 1.04–2.84 and 0.96–2.35, respectively), pancreatic necrosis occurred more frequently in AAP than BAP patients (OR = 1.58, 95% CI 1.08–2.30). Overall, there is a potential association between aetiology and the development and course of AP. HTG-AP is associated with the highest number of complications. Furthermore, AAP is likely to be more severe than BAP or PAP. Greater emphasis should be placed on determining aetiology on admission.

## Introduction

Acute pancreatitis (AP) is a sudden inflammatory disease of the pancreas. In the last 20 years, the incidence of the disease has increased by more than 20%^1,2^. Nowadays, AP is one of the most common reasons for hospitalization in case of gastrointestinal diseases^3^.

Gallstones represent the main aetiological background of AP globally (42%), which are diagnosed by imaging techniques and liver function tests^4^. Gallstone-related or biliary AP (BAP) occurs twice as often as alcohol-induced AP (AAP)^4^. AAP is caused by regular, excessive alcohol consumption usually with a clinical history of > 5 years and > 50–100 g/day^5^. Hypertriglyceridaemia (HTG) with serum triglyceride concentrations > 11.3 mM is the third most common (9%) known aetiological factor of the disease^6–8^. Less frequent causes of AP include endoscopic retrograde cholangiopancreatography (ERCP), hypercalcaemia, pancreas divisum, tumours, genetic polymorphisms and drugs^9^. To date, no standardized diagnostic criteria exist for post-ERCP AP (PAP). The guidelines recommended by Cotton et al*.*^10^ are most commonly applied, which suggest PAP to be diagnosed if pancreatitis develops within 24 h after the procedure.

Based on the Revised Atlanta Classification (RAC), AP severity can be categorized into three groups: mild, moderately severe and severe^11^. Although the majority of cases are mild with a self-limiting course^11^, the mortality rate of severe AP can reach 30% which underlies the desperate need of finding proper treatment^12^. Organ failure (OF) is the most important determinant of this classification system^11^. Patients with mild AP have no organ dysfunction and usually recover within a week. Moderately severe AP resolves slower and might require interventions because of the presence of transient organ failure (TOF, < 48 h). Severe AP results in persistent organ failure (POF) which lasts > 48 h. Multiple organ failure (MOF) is defined as failure of two or more organ systems, which can be transient or persistent^13^. The three extrapancreatic organs most commonly affected by AP are the lungs, the heart and the kidneys^11^. Approximately 25% of AP patients develop severe complications and have to be admitted to an intensive care unit (ICU)^14^. Local complications can also occur in cases of moderately severe and severe AP, which include acute peripancreatic fluid collections, pancreatic pseudocysts, acute necrotic collections and walled-off necrosis^11^. About 20% of patients experience recurrent AP (RAP), which refers to a clinical condition defined by repeated episodes of AP^15^. 10% of AP patients with a single episode and 36% with RAP progress to chronic pancreatitis (CP)^15^. The risk of progression to CP increases with excessive alcohol consumption, smoking and male gender. 5% of CP patients develop pancreatic cancer^16^.

Although there are several risk factors, it is difficult to predict which patient will develop mild, moderately severe or severe AP. To date, numerous clinical studies have investigated the effect of aetiology on AP progression. However, to the best of our knowledge, there have been no efforts to summarize clinical data on how various aetiological backgrounds affect the severity and course of AP. Consequently, this study was undertaken to reveal the impact of the above-mentioned aetiologies (HTG-AP, AAP, BAP, PAP) by performing thorough literature search and meta-analysis on available clinical data.

## Methods

This systematic review and meta-analysis followed the recommendations of Stroup et al.^17^ and was conducted in line with the Preferred Reporting Items for Systematic Reviews and Meta-Analyses guidelines^18^ (Supplementary Table S1). The analysis was based on the Problem, Intervention, Comparison intervention and Outcome (PICO) model^18^ as follows: AP patients with alcoholic, biliary, hypertriglyceridaemic and post-ERCP aetiologies were compared in order to examine the effect of aetiology on disease outcomes. Primary outcome was severity, secondary outcomes were POF, MOF, TOF, ICU admission, recurrence rate, mortality, pancreatic necrosis, pulmonary failure (PUF), renal failure, length of hospital stay (LOS), pseudocyst, fluid collection, and systematic inflammatory response syndrome (SIRS).

The protocol for the meta-analysis was registered in the PROSPERO database on 15/05/2018 (https://www.crd.york.ac.uk/PROSPERO/, ID: CRD42018093574).

### Search strategy

Literature search was conducted in the electronic databases Embase and Pubmed from publication date 01/01/2012 to 31/05/2020. The reason for the start date is that the RAC was introduced in 2012, which provides the most accepted and widespread criteria for determining AP severity. The following search query was used for Embase: (alcohol* OR ethanol* OR biliary OR gallstone OR cholelithiasis OR 'post-ercp' OR 'post ercp' OR idiopathic OR triglyceride OR hypertriglyceridemia OR hyperlipidemia OR severity OR severe OR mild OR moderate) AND acute AND pancreatitis NOT ('conference abstract'/it OR 'review'/it)) AND (2012:py OR 2013:py OR 2014:py OR 2015:py OR 2016:py OR 2017:py OR 2018:py OR 2019:py OR 2020:py). In Pubmed, the following search terms were applied: (alcohol* OR ethanol* OR biliary OR gallstone OR cholelithiasis OR "post-ercp" OR "post ercp" OR idiopathic OR triglyceride OR hypertriglyceridemia OR hyperlipidemia OR severity OR severe OR mild OR moderate) AND acute AND pancreatitis NOT Review[ptyp] NOT Case Reports[ptyp]. The search was restricted to studies written in English or in Hungarian.

### Eligibility criteria

All randomised trials, retrospective and prospective cohort studies were included that involved adult patients with AP and relevant data (primary and secondary outcomes) are categorized according to the aetiology of the disease. Four major disease backgrounds were included: alcohol abuse, HTG, biliary disease and post-ERCP. Articles that studied only one aetiological group or compared one aetiological group with another group called others or non-… (e.g. alcohol vs. non-alcohol) were excluded. Non-human studies or articles with data from patients younger than 18 years of age were not included. In case of cohort overlap between studies, only the most recent study was included unless a prior study had higher quality.

When assessing AP severity, only studies were included where severity was defined according to the RAC, because in this case it was crucial to present a consistent and clear definition for the analysis. Articles were also excluded if only one or two of the three severity groups were analysed. Both local complications and OFs could lead to serious conditions and death which are characteristic features of moderately severe and severe AP. Therefore, these two groups were combined in our study, and are referred to as “non-mild” disease forms and compared to the mild group. In cases of outcomes other than severity, using only the RAC was not in the criteria.

### Study selection and data extraction

Titles and abstracts of publications were screened independently by two review authors (E.R.B. and G.F.) to identify studies that potentially meet inclusion criteria. The full texts of these potentially eligible studies were independently assessed for eligibility by the same two review authors. Disagreement between reviewers was resolved by discussion with other two colleagues (L.K. and Z.R.). E.R.B. and G.F. independently extracted study characteristics (author, title, journal, study location, inclusion period, number of centres involved, type of study, number of participants) and outcome data (severity, POF, MOF, TOF, ICU admission, recurrence rate, mortality, pancreatic necrosis, PUF, renal failure, LOS, pseudocyst, fluid collection, SIRS), which were recorded on a standardized Microsoft Excel spreadsheet. Discrepancies were resolved by discussion.

### Quality assessment

Methodological quality of the articles was assessed by applying the Quality In Prognosis Studies (QUIPS) tool^19^ (Supplementary Table S2). This considers the following domains: study participation, study attrition, prognostic factor measurement, outcome measurement, study confounding, and statistical analysis and reporting. All domains were scored by three individual researchers (E.R.B., G.F., L.K., each article was assessed by at least two of them). The overall risk of bias was considered:

- low if < 3 domains were rated a moderate risk of bias and all others were rated a low risk of bias,
- moderate if ≥ 3 domains were rated a moderate risk of bias and all others were rated a low risk of bias,
- high if ≥ 1 domain was rated a high risk of bias, irrespective of all other domains.

Consensus was reached after classification by the individual researchers.

### Data analyses

Statistical analysis was performed with Stata 11 SE (StataCorp LLC, College Station, TX, USA). The investigated aetiologies were analysed pairwise. Odds ratios (ORs) calculated from patient numbers were used to compare outcomes in different aetiologic groups. ORs were pooled using the random effects model with the DerSimonien–Laird estimation and displayed on forest plots. Summary OR estimation, p value and 95% confidence interval (CI) were calculated. P < 0.05 was considered as significant difference from summary OR = 1. BAP was defined as primary reference group, the other aetiologies were ranked in the following order: AAP, HTG-AP, PAP.

Statistical heterogeneity was analysed using the I^2^ statistic and the chi-square test to acquire probability values; p < 0.1 was defined to indicate significant heterogeneity. The small-study effect (in case of comparisons with at least 10 articles) was visually investigated on funnel plots and was also confirmed by Egger’s test. Sensitivity analysis was performed to examine the robustness of our results.

## Results

### Study selection

**Figure 1:**
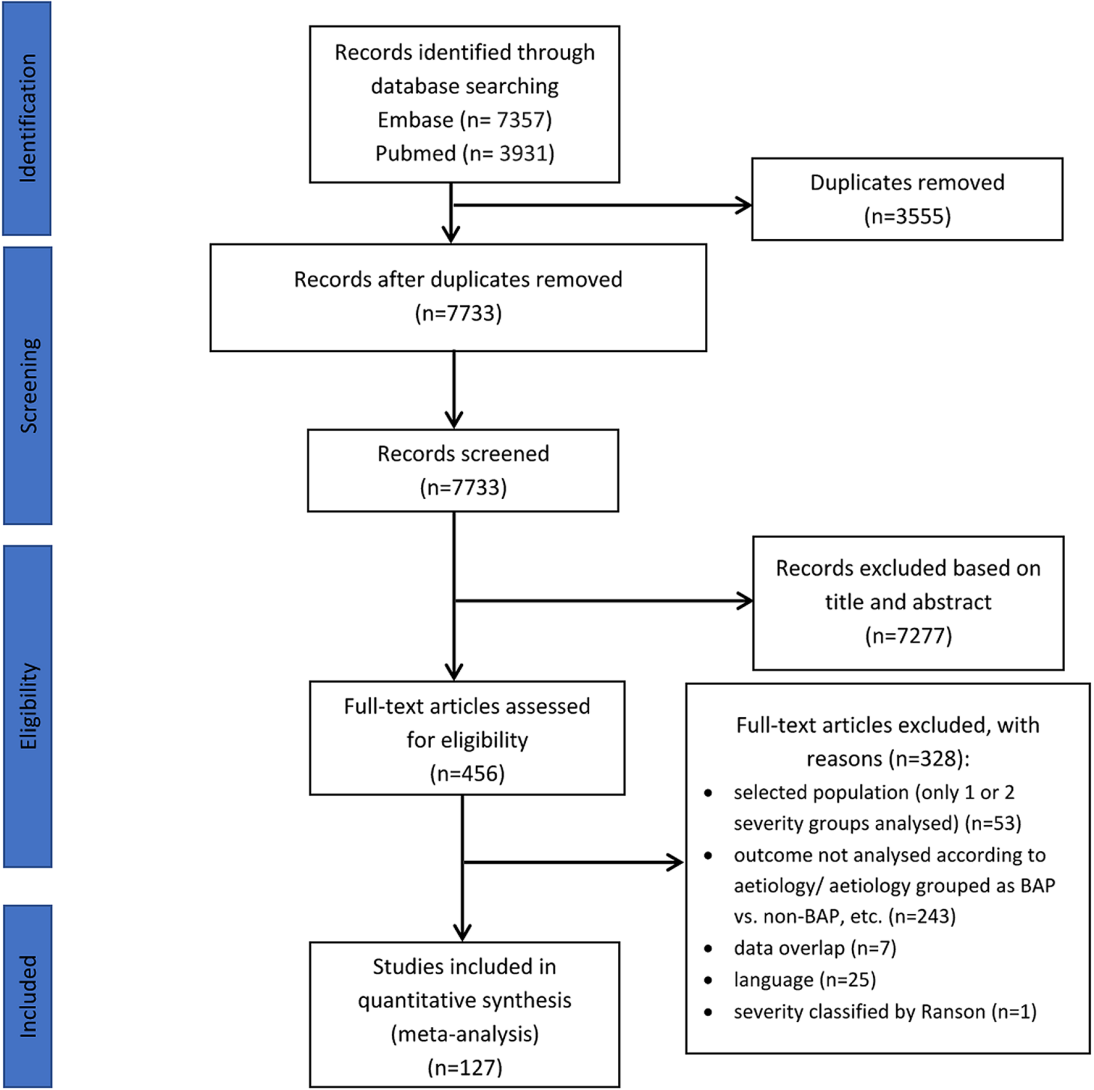
PRISMA 2009 flow diagram for identification of relevant articles.

The search strategy identified 11,288 records. After removing duplicates 7733 articles were retrieved. Out of these, 456 records seemed to be relevant to the study question based on screening by title or abstract. After assessing the articles in full text, 328 records had to be excluded with different reasons (see details in Fig. 1). Finally, 127 publications fulfilled the eligibility criteria.

### Characteristics of studies included

The majority of the included cohort studies (108 out of 128) collected data from the 2010′s. Our meta-analysis contains 102 single^20–121^ and 23 multicentre studies^122–144^. In two cases, there were no relevant data regarding the number of centres involved^145,146^. Sample sizes ranged from 11 to 1,165,777. Only the data of the four types of AP (AAP, BAP, HTG-AP, PAP) were analysed. Detailed characteristics of the included studies are provided in Supplementary Table S3. During quality assessment, we evaluated patient selection, comparability of the groups, and outcome data, which are presented in Supplementary Table S4.

### Risk of bias assessment

According to the QUIPS checklist, most of the included studies had an overall moderate risk of bias (80, 63%; Supplementary Figure S1). 30 studies (23.4%) had low and 17 (13.3%) had high risk of bias. High risk was mainly due to the confounding factors which showed significant difference between the analysed aetiological groups. Moderate risk of bias resulted mainly from „Study confounding” and „Statistical Analysis and Reporting”, furthermore „Prognostic factor measurement” was also missing in a relatively high number (66.9%) of included studies. A detailed analysis can be found in Supplementary Table S4.

### Clinical outcomes

#### Severity

**Figure 2:**
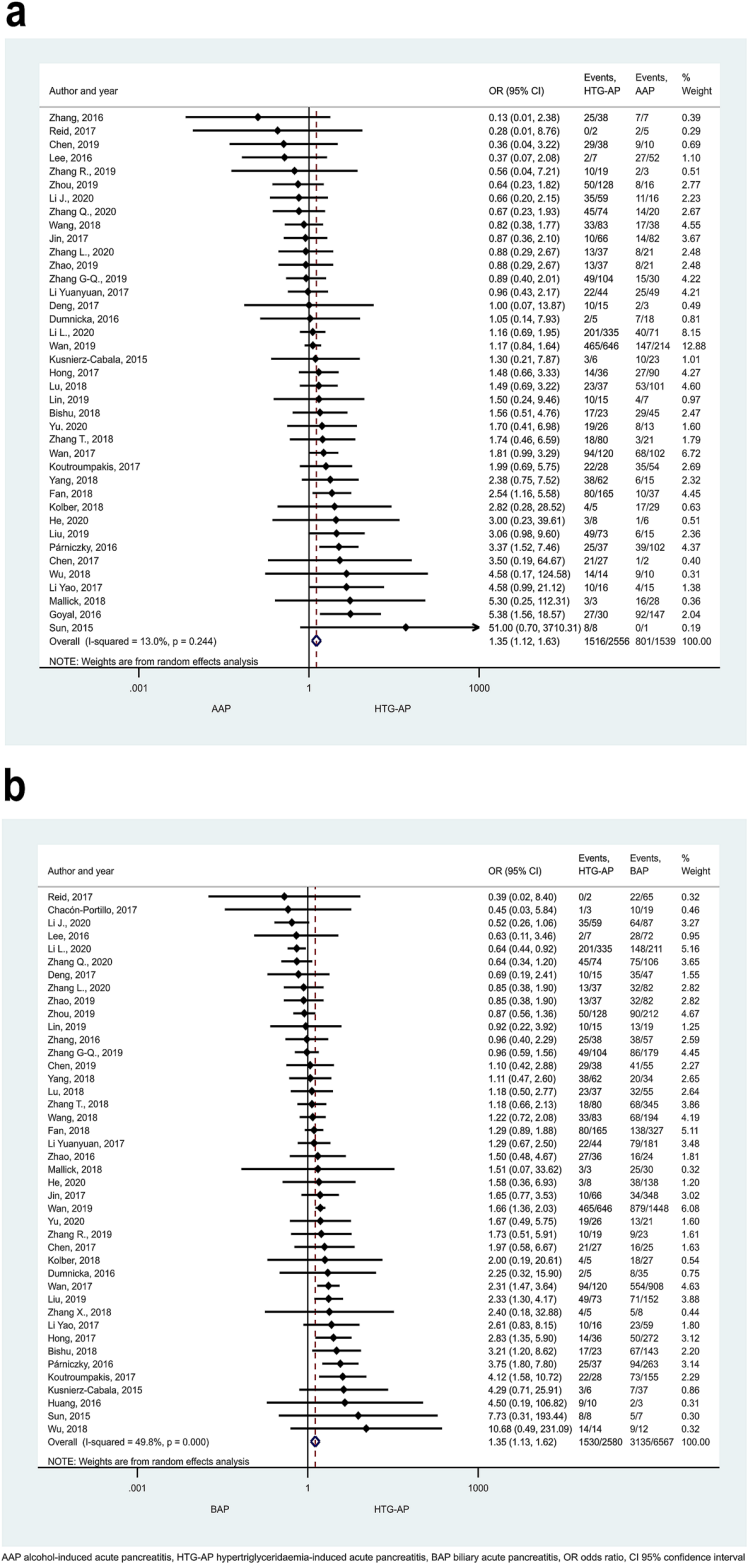
Forest plot showing the comparison of disease severity in (**A**) HTG-AP and AAP, p = 0.001; (**B**) HTG-AP and BAP, p = 0.001. Filled diamonds represent the ORs derived from the articles analysed. Horizontal bars represent CI. Empty diamond shows the overall OR (middle of the diamond and CIs are the edges) for non-mild (moderately severe and severe groups based on the Revised Atlanta Classification) disease. Heterogeneity of the results was presented by I-square and p value.

**Figure 3:**
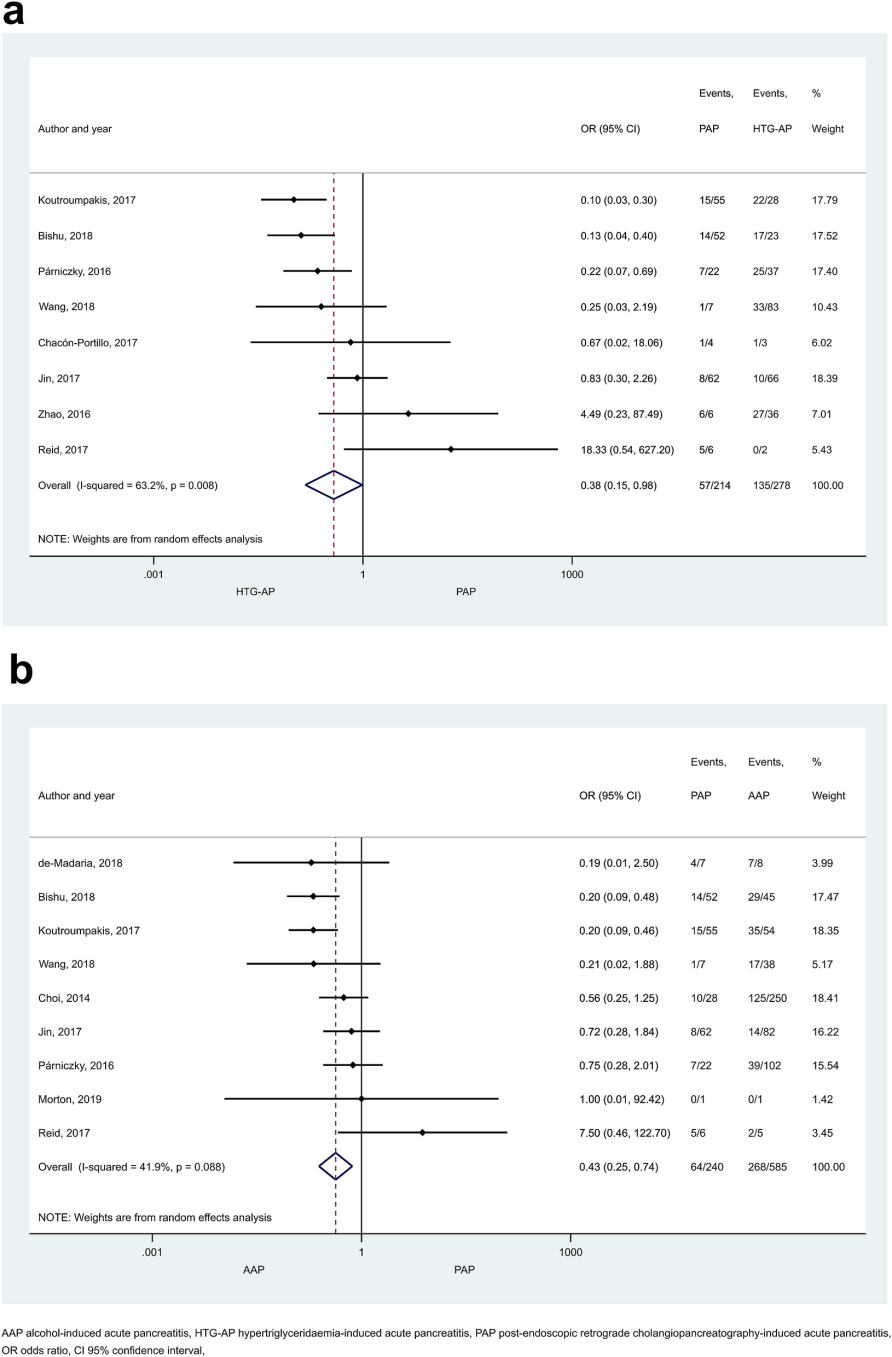
Forest plot showing the comparison of disease severity in (**A**) HTG-AP and PAP, p = 0.045; (**B**) AAP and PAP, p = 0.002. Filled diamonds represent the ORs derived from the articles analysed. Horizontal bars represent CI. Empty diamond shows the overall OR (the middle of the diamond, CIs are the edges) for non-mild disease.

**Figure 4:**
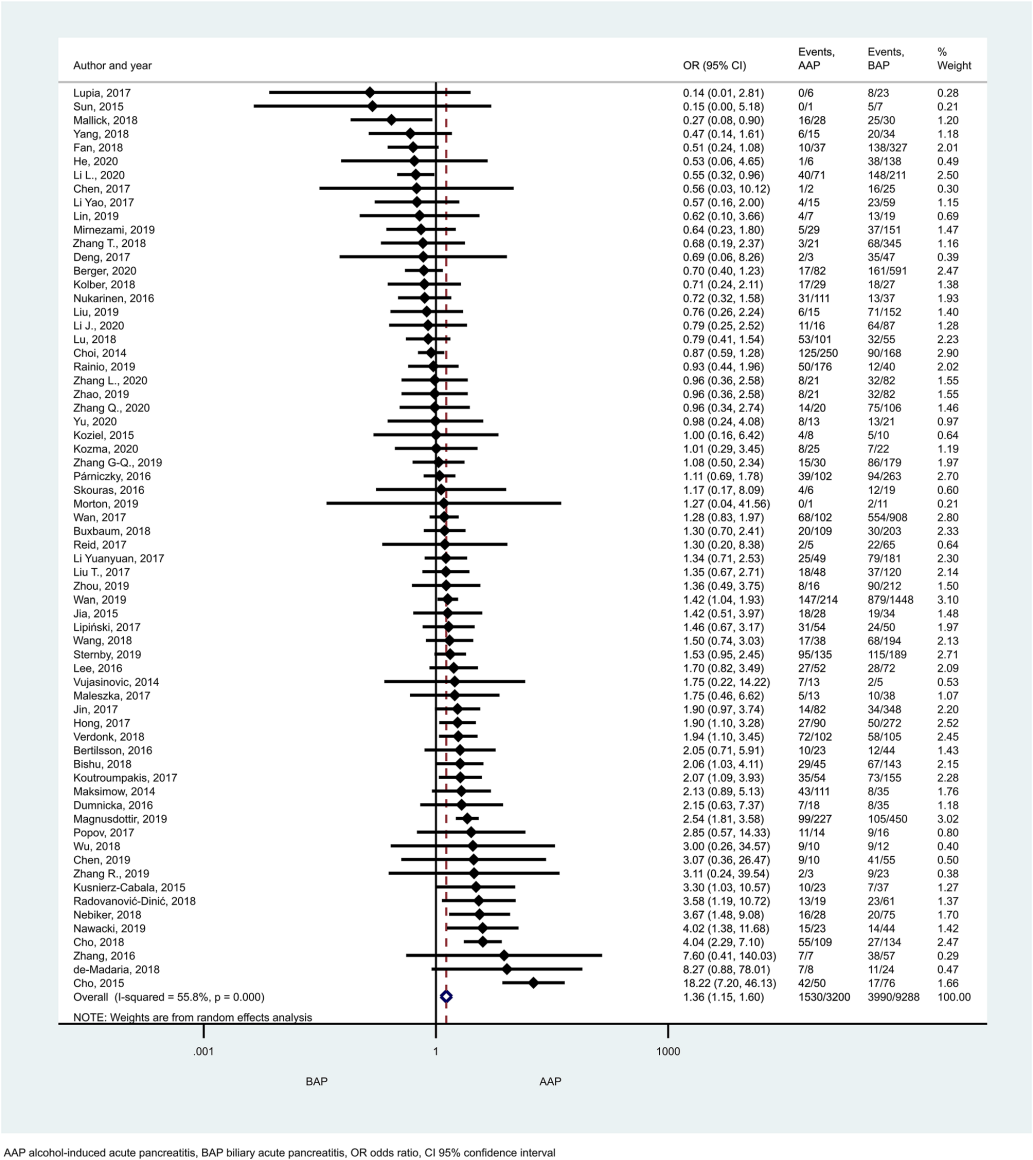
Forest plot showing the comparison of disease severity in AAP and BAP, p < 0.001. Filled diamonds represent the ORs derived from the articles analysed. Horizontal bars represent CI. Empty diamond shows the overall OR (middle of the diamond, CIs are the edges) for non-mild disease.

HTG proved to induce non-mild AP in a significantly higher number of cases than the other aetiological factors (Figs. 2, 3). ORs of non-mild cases in HTG-AP were 1.35 [CI 1.12–1.63] and 1.35 [CI 1.13–1.62] vs. AAP and BAP, respectively (Fig. 2a, b). PAP also appeared to be significantly less severe compared to HTG-AP (Fig. 3a; OR: 0.38 [CI 0.15–0.98]) or AAP (Fig. 3b; OR: 0.43 [CI 0.25–0.74]), while no significant difference could be detected between the severities of BAP and PAP (Supplementary Figure S2]). Alcoholic aetiology significantly increased AP severity compared to biliary-related events (Fig. 4; OR: 1.36 [CI 1.15–1.60]). We found heterogeneity in the comparison of HTG-AP vs. BAP, HTG-AP vs. PAP, AAP vs. BAP, PAP vs. AAP, BAP vs. PAP (Figs. 2b, 3, 4 and Supplementary Figure S2). No signs of small-study effect were detected in any comparison (Supplementary Figure S3).

#### Organ failures, intensive care unit admission, and systematic inflammatory response syndrome

**Figure 5:**
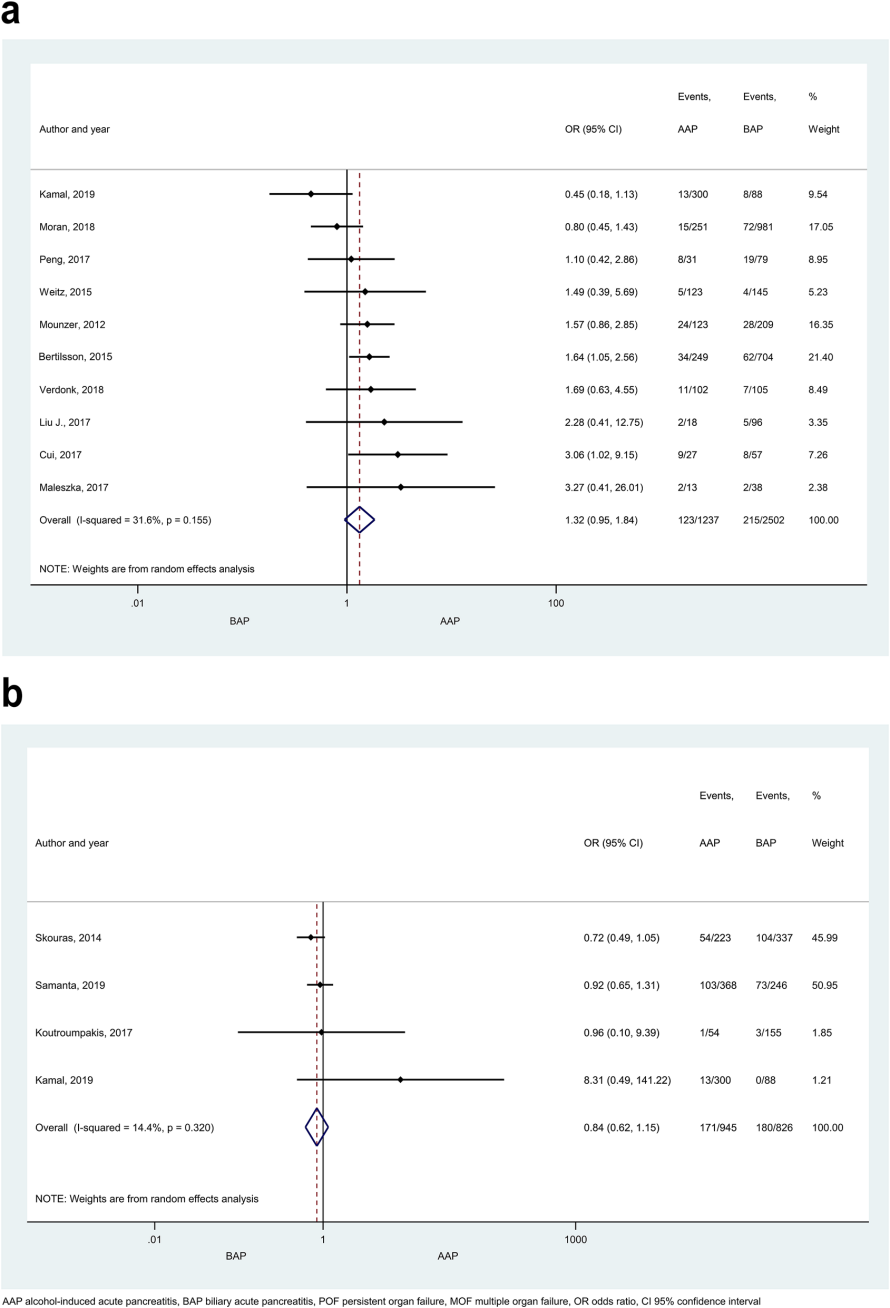
Forest plot showing the effect of different disease aetiologies on POF and MOF. The effects of BAP vs. AAP on (**A**) POF, p = 0.102; and (**B**) MOF, p = 0.284. Filled diamonds represent the ORs derived from the articles analysed. Horizontal bars represent CI. Empty diamond shows the overall OR (the middle of the diamond, CIs are the edges).

No significant difference was found in POF between any aetiological groups (AAP vs. BAP, HTG-AP vs. AAP, HTG-AP vs. BAP; Fig. 5a; Supplementary Figure S4). No signs of small-study effect were found in POF in the comparison of AAP vs. BAP (Supplementary Figure S5a). There were no significant differences in the occurrences of MOF, TOF or renal failure between AAP and BAP (Fig. 5b and Supplementary Figures S6a,b, respectively). PUF occurred more frequently in HTG-AP patients compared to BAP (Supplementary Figure S7a; OR: 2.39 [CI 1.06–5.39]), while AAP and BAP patients did not differ in this respect (Supplementary Figure S7b). The frequency of ICU admission was similar in AAP and BAP patients (Supplementary Figure S8). More AAP patients developed SIRS than PAP patients (Supplementary Figure S9a, OR: 0.40 [CI 0.21–0.77]. The rate of SIRS did not differ when comparing other patient groups (Supplementary Figure S9b,c). Heterogeneity was found in the comparison of renal failure, PUF and ICU admission between AAP and BAP (Supplementary Figures S6b, S7b, S8).

#### Recurrence rate and length of hospital stay

Recurrence rate was significantly higher in AAP vs. BAP patients (Supplementary Figure S10a; OR: 2.98 [CI 2.22–4.01]) and in HTG-AP vs. BAP patients (Supplementary Figure S10b; OR: 2.69 [CI 1.55–4.65]). However, AP did not reoccur more frequently due to alcoholic aetiology than HTG or post-ERCP (Supplementary Figure S11a,b). Recurrence rate was also similar in BAP and PAP patients (Supplementary Figure S11c). Patients of the analysed aetiologies were hospitalized for a similar length of time (Supplementary Figure S12a,b). We found heterogeneity in the comparison of all cases of LOS and all cases of recurrence rate, except for the comparison between AAP and HTG-AP (Supplementary Figure S11a). No signs of small-study effect could be detected in case of recurrence rate or LOS (Supplementary Figure S5b,c).

#### Mortality and pancreatic necrosis

**Figure 6:**
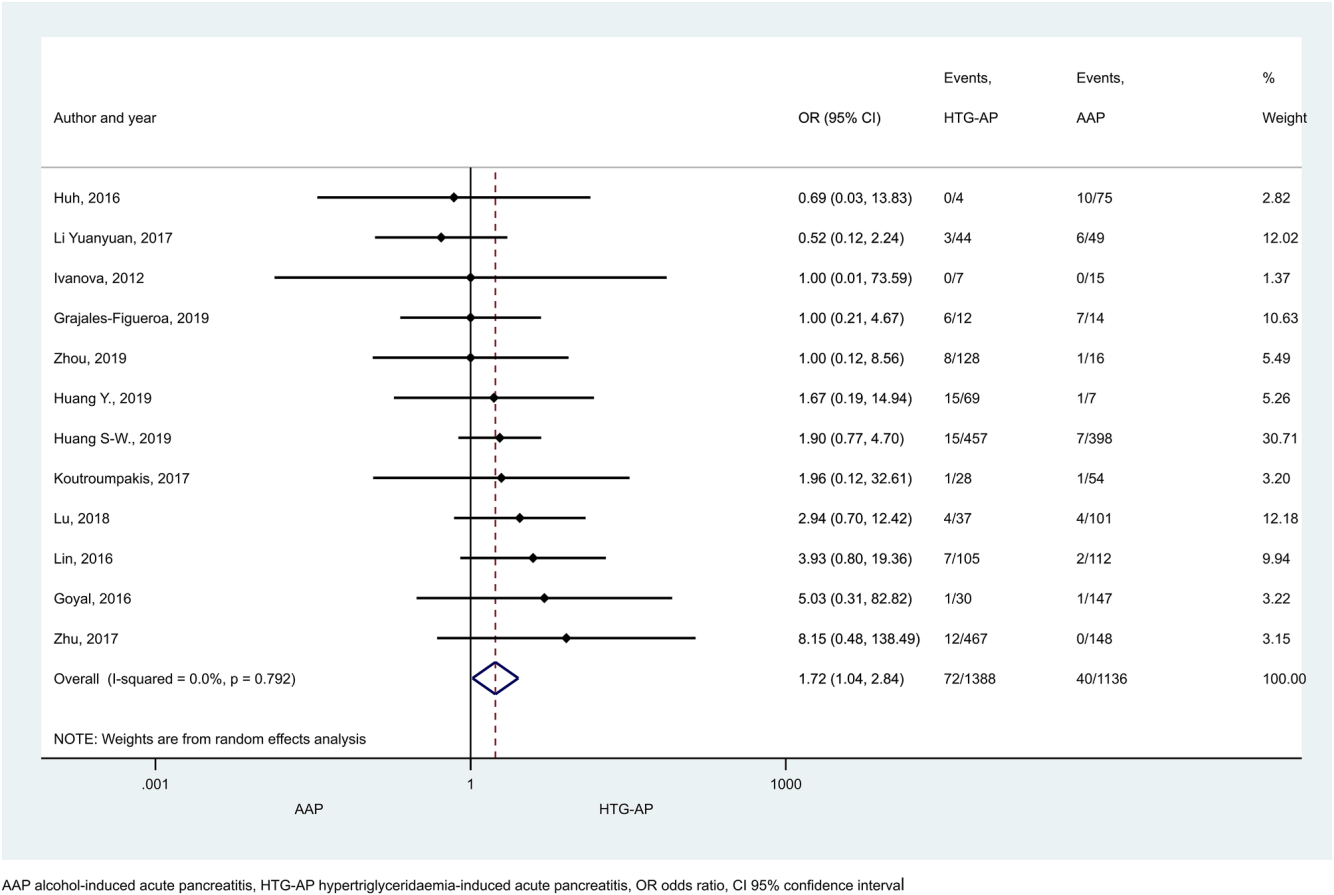
Forest plot showing the effect of HTG-AP and AAP on mortality, p = 0.034. Filled diamonds represent the ORs derived from the articles analysed. Horizontal bars represent CI. Empty diamond shows the overall OR (the middle of the diamond, CIs are the edges).

**Figure 7:**
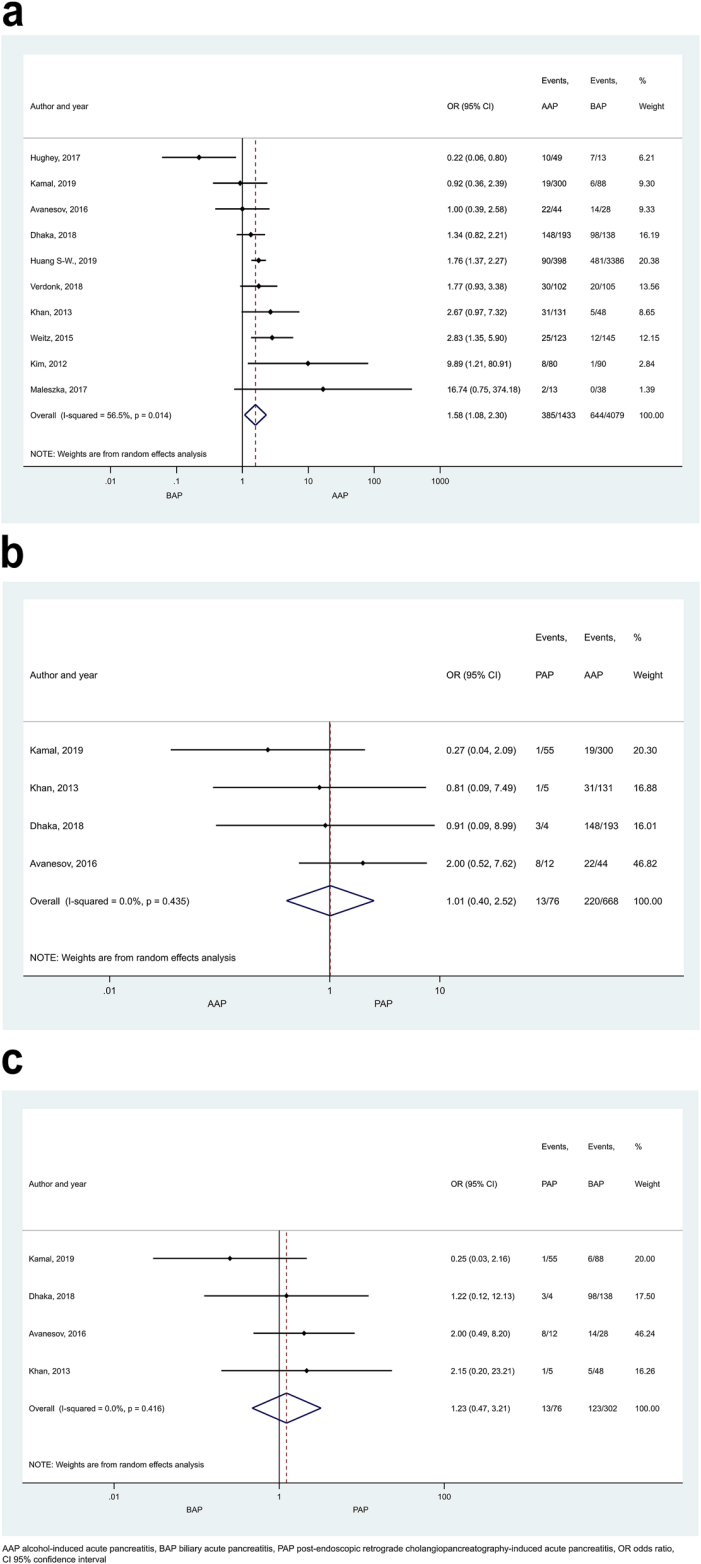
Forest plot showing the effects of different disease aetiologies on pancreatic necrosis. The effects of (**A**) BAP vs. AAP, p = 0.019; (**B**) AAP vs. PAP, p = 0.982; (**C**) BAP vs. PAP, p = 0.674. Filled diamonds represent the ORs derived from the articles analysed. Horizontal bars represent CI. Empty diamond shows the overall OR (the middle of the diamond, CIs are the edges).

Mortality rate proved to be significantly higher in HTG-AP than in AAP (Fig. 6; OR: 1.72 [CI 1.04–2.84]), but no statistical difference was found between any other patient groups (Supplementary Figures S13 and S14). In the comparison of AAP and BAP a large proportion of patients came from one study contributing 1,165,777 subjects (accounting for 12.76% weight, Supplementary Figure S13a). However, sensitivity analysis showed that the results remained similar when this study was excluded (OR = 0.96 [CI 0.75–1.23]; Supplementary Figure S15). Pancreatic necrosis was reported more often in AAP than BAP patients (Fig. 7a, OR = 1.58 [CI 1.08–2.30]). No significant difference was detected in any other comparisons regarding necrosis (Fig. 7b,c). Heterogeneity was found in the comparison of mortality rate between BAP and HTG-AP, AAP and BAP (Supplementary Figure S13), and in case of necrosis when AAP and BAP groups were compared (Fig. 7a). We found signs of the small-study effect in case of mortality in the comparison of AAP and BAP (Supplementary Figure S16).

#### Pseudocyst, fluid collection

There was no significant difference in the presence of fluid collection or pseudocysts in the available comparisons (Supplementary Figure S17). We found heterogeneity in case of fluid collection (AAP vs. BAP) and pseudocyst (HTG-AP vs BAP).

## Discussion

This is the first detailed meta-analysis investigating the relationship between different aetiologies (alcohol abuse, biliary, HTG, post-ERCP) and the course of AP. Our study revealed that the prevalence of severe and moderately severe (non-mild) disease forms was highest in case of HTG-AP which was followed by AAP, BAP and PAP (Table 1). Due to the large number of included articles and patients, our results have strong evidence in case of the severity outcome. These are also in accordance with our earlier observations^147^ and the data of Wang et al.^148^. However, previously we compared the severe disease category to moderately severe and mild groups, which has less relevance than merging the severe and moderately severe groups as we did in the current study. Furthermore, our previous study and that of Wang et al.^148^ compared the characteristics and outcome of HTG-AP to non-HTG-AP patients, without classifying non-HTG-AP group any further according to aetiology. Importantly, non-HTG-AP group is rather heterogenous and it cannot be decided whether all aetiological subgroups included in non-HTG-AP are less severe than HTG-AP or some of the subgroups are basically more severe than HTG-AP but due to other much milder subtypes, HTG-AP turns out to be the most severe disease form. In addition, non-HTG-AP included all aetiologies (“idiopathic” and “other” as well) except HTG-AP, while we only analysed well-defined and clear aetiologies. Therefore, the current meta-analysis provides a more refined picture of the outcomes. Wang et al.^148^ evaluated AP severity using the APACHE-II scoring system, but this does not specifically define AP severity.

**Table 1:** Summary of the results of our study.

Severity	HTG-AP > AAP > BAP ≸ PAP
POF	AAP ≸ BAP HTG-AP ≸ [AAP/BAP]
MOF	AAP ≸BAP
TOF	AAP ≸BAP
PUF	HTG-AP > BAP ≸ AAP
Renal failure	AAP ≸BAP
ICU admission	AAP ≸ BAP
SIRS	BAP ≸ AAP > PAP BAP ≸ PAP
Recurrence rate	[HTG-AP/AAP] > BAP HTG-AP ≸ AAP PAP ≸ [AAP/ BAP]
Mortality	HTG-AP > AAP [HTG-AP/AAP/PAP] ≸ BAP AAP ≸ PAP
Necrosis	AAP > BAP PAP ≸ [AAP/BAP]
Pseudocyst	HTG-AP ≸ BAP AAP ≸ BAP
LOS	[HTG-AP/AAP] ≸ BAP

*AAP* alcohol-induced acute pancreatitis, *BAP* biliary acute pancreatitis, *HTG-AP* hypertriglyceridaemia-induced acute pancreatitis, *ICU* intensive care unit, *LOS* length of hospital stay, *MOF* multiple organ failure, *PAP* post endoscopic retrograde cholangiopancreatography-induced acute pancreatitis, *POF* persistent organ failure, *PUF* pulmonary failure, *SIRS* systematic inflammatory response syndrome, *TOF* transient organ failure.

Statistically siginificant difference (p < 0.05) was presented with < ; ≸ shows no significant difference.

In our study, no difference could be observed between any aetiological groups in POF (Table 1). Although HTG-AP and AAP exhibited the most severe forms of AP from an aetiologic point of view, the data of POF does not support it. This can be explained by the fact that POF associated with AP was assessed in HTG-AP patients only in three of the articles included in our meta-analysis^38,77,90^. In the study of Wang et al.^148^ POF was most commonly observed in HTG-AP, which is in accordance with our results for severity. Although several articles evaluate characteristic features of severe AP such as POF, they focus exclusively on severe AP patients. For this reason, these could not be utilized in our analysis.

MOF is another distinctive feature of severe and moderately severe AP. In the current study, no significant difference could be detected in MOF between any of the analysed groups. Similarly, in a previous study, we did not find differences in MOF among HTG-AP vs. non-HTG-AP patients^147^. Tai et al.^149^ also found a higher risk for the severe form of AP in HTG-AP patients compared to BAP. They diagnosed MOF more frequently in BAP patients, however, there was no difference in single OFs (renal, heart, pulmonary).

AP patients with systemic complications eventually end up in ICU. In case of this outcome, only one comparison could be performed: no significant difference was found between AAP and BAP (Table 1), which is supported by our previous findings^147^.

The 27% recurrence rate of AP in the 1990s^150^ has nowadays decreased to about 20%^151^, which could be explained by better diagnosis and treatment after the first attack. In our study, alcoholic and hypertriglyceridaemic aetiologies caused more AP recurrence than biliary, while the repeated hospitalization for AAP and HTG-AP patients was similar. Tai et al. also found higher recurrence rate of HTG-AP than BAP^149^. Other studies drew the conclusion that alcohol is the most frequent aetiological factor for recurrent AP^150,151^. Suchsland et al.^151^ analysed the risk factors for readmittance in AP, most of which were related to alcohol abuse, so these patients have a higher risk for disease recurrence after discharge. In case of BAP, delayed cholecystectomy could be responsible for recurrence^152,153^.

Our study has shown that HTG-AP led to significantly higher mortality rate than AAP. However, no significant difference could be detected between the other aetiological groups. BAP used to have a higher mortality than AAP; however, this rate has decreased in the last decade due to improved supportive care^154^. Several studies have reported that mortality rate was not influenced by aetiological factors^155,156^. Other studies stated that HTG-AP did not cause significantly higher mortality rate, even though it led to higher severity and complication rates compared to other aetiological factors^157,158^. Wang et al.^148^ concluded that HTG-AP caused higher mortality rate than non-HTG-AP, while Kiss et al.^147^ did not find significant difference in this respect. Based on the current study, there is no strong relationship between aetiology of AP and mortality.

HTG carried the greatest risk for non-mild (moderately severe and severe) AP, which was followed by AAP; the least severe disease forms were observed in BAP and PAP. One of the possible pathomechanisms is that lipotoxicity mediated by unsaturated fatty acids contributes to necrosis, OF (eg. cardiovascular diseases) and mortality^159^. Experimental studies also demonstrated that HTG exacerbates the severity of AP^159,160^. Fatty acid administration resulted in elevated intracellular Ca^2+^ levels in pancreatic acinar cells and impaired mitochondrial function^161,162^. HTG-AP is often accompanied by one or more secondary factors (alcoholism, medications, uncontrolled diabetes mellitus, physical inactivity), which can further aggravate the severity of the disease^163–166^. Furthermore, elevated serum chylomicron concentration during HTG increases viscosity, causing reduced blood flow in microvessels and resulting in ischemic conditions. This could be an additional risk factor for a severe form of AP^161,162^.

Determining the exact aetiology of AP may be challenging in some cases. For example, alcohol is not only known as an independent risk factor for AP but can also increase serum TG concentrations, as mentioned before. In addition, mild-to-moderate elevation in TG concentrations can be observed in the early phase of AP, regardless of aetiology^167^. Since TG concentrations can rapidly decrease during fasting state after the diagnosis of AP, the measurement of TG concentrations on (or shortly after) admission is crucial.

The number of events, which refer to positive outcomes in certain aetiologies were relatively high in case of severity (1516 severe events occurred out of 2556 HTG-AP patients in Fig. 2a) and partly in mortality (10,161 events/620,027 BAP patients in Supplementary Figure S13a) and recurrence rate outcomes (1671 events/5254 AAP patients in Supplementary Figure S10a). Smaller number of events (9–97) could be included in the analysis of other outcomes (Table 1). Low event rates can have detrimental influence on the reliability of the results^168,169^. Based on the studies mentioned above, the results of all severity comparisons, mortality and recurrence rates in comparisons of AAP vs. BAP are strongly reliable. Most of the other calculations have lower reliability but there is no precedent to contradict the results of severity.

The current meta-analysis has strengths and limitations that should be noted. The major strengths are the following: we included a large number of articles. Four major aetiologies were analysed, leaving out miscellaneous or idiopathic backgrounds. For the analysis of severity, we only included articles where severity was defined according to the RAC, which provided a clear and consistent base for the comparisons. In addition, we compared mild to moderately severe and severe (“non-mild”) AP groups, which further refined our analysis. The quality of the involved articles determines the value of pooled data. There has been high variability in methodology of the studies which may have unintended effects on the final results and interpretation, study populations were diverse in age and gender, which might cause heterogeneity in aetiologic distribution. Aetiologies were not necessarily defined the same way. Certain outcomes (e.g. necrosis) were only evaluated by a limited number of studies, especially in case of HTG-AP, which may be the reason that no statistically significant difference could be detected between HTG-AP and other aetiologies or no statistical analysis could be performed. One article analysed data from 1975 to 2010, which was only applied for the assessment of recurrence rate. Another article contributed 1,165,777 patients to the analysis, which was only used for the evaluation of mortality. In addition, only articles published in English or Hungarian were included.

## Conclusions

AP is a complex disorder mediated by metabolic, environmental and genetic factors, which can lead to death in the most severe forms. Therefore, clinicians should be more alert for a severe disease course in the at-risk patients. Our observations highlight the importance of disease aetiology. We found association between aetiology and the development and course of AP. HTG proved to carry the highest risk for non-mild (moderately severe and severe) AP, which was followed by AAP; the least severe disease forms were observed in BAP and PAP. It is essential to determine the cause of the disease in time to apply the most appropriate therapy. Based on the results, greater emphasis should be placed on determining aetiology on admission, especially in case of HTG-AP.

## Supplementary information

Supplementary Information

## Abbreviations

AP: Acute pancreatitis
AAP: Alcohol-induced/alcoholic acute pancreatitis
BAP: Biliary acute pancreatitis
CI: 95% Confidence interval
ERCP: Endoscopic retrograde cholangiopancreatography
HTG-AP: Hypertriglyceridaemia-induced acute pancreatitis
HTG: Hypertriglyceridaemia
ICU: Intensive care unit
LOS: Length of hospital stay
MOF: Multiple organ failure
OF: Organ failure
OR: Odds ratio
PAP: Post-endoscopic retrograde cholangiopancreatography-induced acute pancreatitis
POF: Persistent organ failure
PUF: Pulmonary failure
RAC: Revised Atlanta Classification
SIRS: Systematic inflammatory response syndrome
TOF: Transient organ failure
