# Supplementary material for: Assessment of the course of acute pancreatitis in the light of aetiology: a systematic review and meta-analysis
Source: Sci Rep. 2020 Oct 21;10:17936. doi: 10.1038/s41598-020-74943-8 (PMC7578029; doi:10.1038/s41598-020-74943-8)

**Assessment of the course of acute pancreatitis in the light of aetiology: a systematic review and meta-analysis**

Emese Réka Bálint^1^, Gabriella Fűr^1^, Lóránd Kiss^1^, Dávid István Németh^2^, Alexandra Soós^2,4^, Péter Hegyi^2,4^, Zsolt Szakács^2^, Benedek Tinusz^2^, Péter Varjú^5^, Áron Vincze^5^, Bálint Erőss^2^, József Czimmer^2^, Zoltán Szepes^6^, Gábor Varga^7^, Zoltán Rakonczay Jr.^1^

^1^ Institute of Pathophysiology, Medical School, University of Szeged, 1 Semmelweis street, H-6701, Szeged, Hungary

^2^ Institute for Translational Medicine and Szentágothai Research Centre, Medical School, University of Pécs, 20 Ifjúság útja, H-7624, Pécs, Hungary

^3^ MTA-SZTE Momentum Translational Gastroenterology Research Group, 13 Dugonics tér, H-6720, Szeged, Hungary

^4^ Clinical Medicine Doctoral School, University of Szeged, 6 Korányi fasor, H-6720, Szeged, Hungary

^5^ First Department of Medicine, Medical School, University of Pécs, 20 Ifjúság útja, H-7624, Pécs, Hungary

^6^ First Department of Medicine, Medical School, University of Szeged, 8-10 Korányi fasor, H-6720, Szeged, Hungary

^7^ Department of Oral Biology, Semmelweis University, Budapest, 4 Nagyvárad tér, H-1089 Budapest Hungary

Correspondence: [rakonczay.zoltan@med.u-szeged.hu](mailto:rakonczay.zoltan@med.u-szeged.hu)

**Supplementary Tables**

**Supplementary Table S1.** Checklist as the Transparent Reporting System of Systematic Reviews and Meta-Analyses Statement recommend.

| **Section/topic** | **#** | **Checklist item** | **Reported on page #** |
| --- | --- | --- | --- |
| **TITLE** | | |  |
| Title | 1 | Assessment of the course of acute pancreatitis in the light of aetiology: a systematic review and meta-analysis | 1 |
| **ABSTRACT** | | |  |
| Structured summary | 2 | Provide a structured summary including, as applicable: background; objectives; data sources; study eligibility criteria, participants, and interventions; study appraisal and synthesis methods; results; limitations; conclusions and implications of key findings; systematic review registration number. | 1 |
| **INTRODUCTION** | | |  |
| Rationale | 3 | Describe the rationale for the review in the context of what is already known. | 3 |
| Objectives | 4 | Provide an explicit statement of questions being addressed with reference to participants, interventions, comparisons, outcomes, and study design (PICOS). | 4 |
| **METHODS** | | |  |
| Protocol and registration | 5 | Indicate if a review protocol exists, if and where it can be accessed (e.g., Web address), and, if available, provide registration information including registration number. | 5 |
| Eligibility criteria | 6 | Specify study characteristics (e.g., PICOS, length of follow-up) and report characteristics (e.g., years considered, language, publication status) used as criteria for eligibility, giving rationale. | 5-6 |
| Information sources | 7 | Describe all information sources (e.g., databases with dates of coverage, contact with study authors to identify additional studies) in the search and date last searched. | 5 |
| Search | 8 | Present full electronic search strategy for at least one database, including any limits used, such that it could be repeated. | 5 |
| Study selection | 9 | State the process for selecting studies (i.e., screening, eligibility, included in systematic review, and, if applicable, included in the meta-analysis). | 6 |
| Data collection process | 10 | Describe method of data extraction from reports (e.g., piloted forms, independently, in duplicate) and any processes for obtaining and confirming data from investigators. | 6 |
| Data items | 11 | List and define all variables for which data were sought (e.g., PICOS, funding sources) and any assumptions and simplifications made. | 4 |
| Risk of bias in individual studies | 12 | Describe methods used for assessing risk of bias of individual studies (including specification of whether this was done at the study or outcome level), and how this information is to be used in any data synthesis. | 6-7 |
| Summary measures | 13 | State the principal summary measures (e.g., risk ratio, difference in means). | 7 |
| Synthesis of results | 14 | Describe the methods of handling data and combining results of studies, if done, including measures of consistency (e.g., I^2^) for each meta-analysis. | 7 |
| Risk of bias across studies | 15 | Specify any assessment of risk of bias that may affect the cumulative evidence (e.g., publication bias, selective reporting within studies). | Suppl. Table S4, Suppl. Figure S1 |
| Additional analyses | 16 | Describe methods of additional analyses (e.g., sensitivity or subgroup analyses, meta-regression), if done, indicating which were pre-specified. | sensitivity analysis, Suppl. Figure S15 |
| **RESULTS** | | |  |
| Study selection | 17 | Give numbers of studies screened, assessed for eligibility, and included in the review, with reasons for exclusions at each stage, ideally with a flow diagram. | Figure 1 |
| Study characteristics | 18 | For each study, present characteristics for which data were extracted (e.g., study size, PICOS, follow-up period) and provide the citations. | 8 |
| Risk of bias within studies | 19 | Present data on risk of bias of each study and, if available, any outcome level assessment (see item 12). | Suppl. Table S4; Suppl. Figure S1 |
| Results of individual studies | 20 | For all outcomes considered (benefits or harms), present, for each study: (a) simple summary data for each intervention group (b) effect estimates and confidence intervals, ideally with a forest plot. | Figures 2-7, Suppl. Figures S2, S4, S6-S14, S17 |
| Synthesis of results | 21 | Present results of each meta-analysis done, including confidence intervals and measures of consistency. | 7-10 |
| Risk of bias across studies | 22 | Present results of any assessment of risk of bias across studies (see Item 15). | Suppl. Table 4; Suppl. Figure 1 |
| Additional analysis | 23 | Give results of additional analyses, if done (e.g., sensitivity or subgroup analyses, meta-regression [see Item 16]). | sensitivity analysis; Suppl. Figure S15 |
| **DISCUSSION** | | |  |
| Summary of evidence | 24 | Summarize the main findings including the strength of evidence for each main outcome; consider their relevance to key groups (e.g., healthcare providers, users, and policy makers). | 11-14 |
| Limitations | 25 | Discuss limitations at study and outcome level (e.g., risk of bias), and at review-level (e.g., incomplete retrieval of identified research, reporting bias). | 14 |
| Conclusions | 26 | Provide a general interpretation of the results in the context of other evidence, and implications for future research. | 15-16 |
| **FUNDING** | | |  |
| Funding | 27 | Describe sources of funding for the systematic review and other support (e.g., supply of data); role of funders for the systematic review. | 34 |

**Supplementary Table S2.** The quality of the articles in our meta-analysis was assessed by the Quality In Prognostic Stuidies (QUIPS) tool. The following table lists the investigated questions.

| **Variable** | **Bias Domains** | | |
| --- | --- | --- | --- |
|  | 1. **Study Participation** | 1. **Study Attrition** | 1. **Prognostic Factor (PF, in this study: aetiology) Measurement** |
| Optimal study or characteristics of unbiased study | The study sample adequately represents the population of interest. | The study data available (i.e., participants not lost to follow-up) adequately represent the study sample. | Same aetilogies are measured in a similar way and by using appropriate methods for all participants. |
| Prompting items and considerations | a. Adequate participation in the study by eligible persons | a. Adequate response rate for study participants | a. A clear definition or description of aetiology is provided. |
|  | b. Description of the source population or population of interest | b. Description of attempts to collect information on participants who dropped out | b. Method of aetiology measurement is adequately valid and reliable. |
|  | c. Description of the baseline study sample | c. Reasons for loss to follow-up are provided | c. Continuous variables are reported or appropriate cut points are used- (blood level of TG is above 11.3 mmol/L.) |
|  | d. Adequate description of the sampling frame and recruitment | d. Adequate description of participants lost to follow-up | d. The method and setting of measurement of aetiology is the same for all study participants. |
|  | e. Adequate description of the period and place of recruitment | e. There are no important differences between participants who completed the study and those who did not. | e. Adequate proportion of the study sample has complete data for aetiology. |
|  | f. Adequate description of inclusion and exclusion criteria |  | f. Appropriate methods of imputation are used for missing aetiology. |
| **Ratings** |  |  |  |
| High risk of bias | The relationship between the PF and outcome is very likely to be different for participants and eligible nonparticipants. | The relationship between the PF and outcome is very likely to be different for completing and noncompleting participants. | The measurement of the PF is very likely to be different for different levels of the outcome of interest. |
| Moderate risk of bias | The relationship between the PF and outcome may be different for participants and eligible nonparticipants. | The relationship between the PF and outcome may be different for completing and noncompleting participants. | The measurement of the PF may be different for different levels of the outcome of interest. |
| Low risk of bias | The relationship between the PF and outcome is unlikely to be different for participants and eligible nonparticipants. | The relationship between the PF and outcome is unlikely to be different for completing and noncompleting participants. | The measurement of the PF is unlikely to be different for different levels of the outcome of interest. |
|  |  |  | |
| **Variable** | **Bias Domains** | | |
|  | 1. **Outcome Measurement** | 1. **Study Confounding** | 1. **Statistical Analysis and Reporting** |
| Optimal study or characteristics of unbiased study | The outcome of interest is measured in a similar way for all participants.  In case only one outcome is reported, we analysed that. In case of multiple outcomes a hierarchy was set up and only one outcome was analysed in the following order: 1. severity, 2. mortality, 3. POF, 4. recurrence, 5. necrosis, 6. pseudocyst. | a. All important confounders are measured.  (1. age, 2. gender, 3. comorbidities or BMI) | Sufficient presentation of data to assess the adequacy of the analytic strategy- there are at least 5 participants in each patient group. |
| Prompting items and considerations | a. A clear definition of the outcome is provided. | b. Clear definitions of the important confounders measured are provided. | b. Strategy for model building is appropriate and is based on a conceptual framework or model. |
|  | b. Method of outcome measurement used is adequately valid and reliable. | c. Measurement of all important confounders is adequately valid and reliable. | c. The selected statistical model is adequate for the design of the study. |
|  | c. The method and setting of outcome measurement is the same for all study participants. | d. The method and setting of confounding measurement are the same for all study participants. | d. There is no selective reporting of results. |
|  |  | e. Appropriate methods are used if imputation is used for missing confounder data. |  |
|  |  | f. Important potential confounders are accounted for in the study design. |  |
|  |  | g. Important potential confounders are accounted for in the analysis. |  |
| **Ratings** |  |  |  |
| High risk of bias | The measurement of the outcome is very likely to be different related to the baseline level of the PF. | The observed effect of the PF on the outcome is very likely to be distorted by another factor related to PF and outcome. | The reported results are very likely to be spurious or biased related to analysis or reporting. |
| Moderate risk of bias | The measurement of the outcome may be different related to the baseline level of the PF. | The observed effect of the PF on outcome may be distorted by another factor related to PF and outcome. | The reported results may be spurious or biased related to analysis or reporting. |
| Low risk of bias | The measurement of the outcome is unlikely to be different related to the baseline level of the PF. | The observed effect of the PF on outcome is unlikely to be distorted by another factor related to PF and outcome. | The reported results are unlikely to be spurious or biased related to analysis or reporting. |

**Supplementary Table S3.** Characteristics of the studies included in the meta-analysis. Abbreviations: AAP: alcohol-induced acute pancreatitis, BAP: biliary acute pancreatitis, HTG-AP: hypertriglyceridaemia-induced acute pancreatitis, ICU: intensive care unit, LOS: length of hospital stay, MOF: multiple organ failure, PAP: post endoscopic retrograde cholangiopancreatography-induced acute pancreatitis, , POF: persistent organ failure, PUF: pulmonary failure, SIRS: systematic inflammatory response syndrome; TOF: transient organ failure N/A: not available, UK: United Kingdom; USA: United States of America.

| **First author, source, year** | **Country** | **Study design** | **Inclusion period** | **Centre** | **Patient number: used for analysis/ total (male)** | **AP aetiology** | **AP outcomes** |
| --- | --- | --- | --- | --- | --- | --- | --- |
| Avanesov, Eur J Radiol, 2016 | Germany | retrospective | January 2011 - August 2016 | single | 84/102 (73) | AAP, BAP, PAP | necrosis |
| Avanesov, Plos One, 2018 | Germany | retrospective | January 2011-May 2017 | single | 225/164 (150) | AAP, BAP, PAP | recurrence |
| Badhal, Trop Gastroenterol, 2012 | India | prospective | N/A | single | 38/30 (23) | AAP, BAP | mortality |
| Berger, Pancreatology, 2020 | Chile | retrospective | 1 May 2014-30 April 2016 | multi | 962/673 (447) | AAP, BAP | severity, recurrence |
| Bertilsson, Clin Gastroenterol Hepatol, 2015 | Sweden | retrospective | 2003-2013 | single | 953/1457 (772) | AAP, BAP | recurrence, POF, mortality |
| Bertilsson, Dig Dis Sci, 2016 | Sweden | prospective | April 2012 - June 2014 | single | 67/92 (51) | AAP, BAP | severity |
| Bishu, Pancreas, 2018 | USA | prospective | 2003-2014 | single | 263/357 (178) | AAP, BAP, HTG-AP, PAP | severity |
| Bogdan, Pol Przegl Chir, 2012 | Poland | retrospective | 1 January 2005- 31 December 2010 | single | 298/226 (208) | AAP, BAP | recurrence |
| Bosques-Padilla, Am J Med Sci., 2015 | Mexico | prospective | June 2008- August 2010 | single | 191/184 (73) | AAP, BAP, HTG-AP, PAP | SIRS |
| Buxbaum, Am J Gastroenterol, 2018 | USA | prospective | March 2015-March 2017 | single | 312/439 (232) | AAP, BAP | recurrence, severity |
| Castoldi, Dig Liver Dis, 2013 | Italy | retrospective | N/A | multi | 475/1173 (592) | AAP, BAP | recurrence |
| Cavestro, Dig Liver Dis, 2015 | Italy | prospective | July 2002 - December 2011 | single | 138/196 (125) | AAP, BAP | recurrence |
| Ćeranić, Bosn J Basic Med Sci, 2019 | Slovenia | prospective | 1 May 2012-31 January 2015 | single | 96/80 (59) | AAP, BAP | recurrence |
| Chacón-Portillo, Rev Invest Clin, 2017 | Mexico | prospective | March 2015 - October 2015 | single | 27/27 (10) | BAP, HTG-AP, PAP | severity |
| Chen, Dig Dis Sci, 2019 | China | prospective | August 2016-September 2018 | single | 113/103 (69) | AAP, BAP, HTG-AP | severity |
| Chen, Pancreas, 2013 | USA | N/A | 1998 - 2007 | multi | 1,165,777/2,242,73 (645,433) | AAP, BAP | mortality |
| Chen, Pancreatology, 2017 | China | N/A | March 2015 - March 2016 | single | 54/57 (36) | AAP, BAP, HTG-AP | severity |
| Cho, BMC Gastroenterol, 2015 | Republic of Korea | retrospective | January 2011 - January 2013 | single | 126/126 (81) | AAP, BAP | mortality, severity |
| Cho, BMC Gastroenterol, 2018 | Republic of Korea | prospective | March 2014-September 2016 | single | 243/243 (166) | AAP, BAP | ICU, mortality, severity |
| Cho, Scand J Gastroenterol, 2020 | Republic of Korea | retrospective | January 2009-December 2014 | single | 617/486 (380) | AAP, BAP | recurrence |
| Choi, Pancreatology, 2014 | Republic of Korea | retrospective cohort (prospectively maintained database) | January 2006 - January 2013 | single | 446/553 (342) | AAP, BAP, PAP | severity |
| Cui, Dig Dis Sci, 2014 | Korea | prospective | January 2011- July 2012 | multi | 302/86 (195) | AAP, BAP | pseudocyst, fluid collection |
| Cui, J Crit Care, 2017 | China | retrospective | January 2014 -January 2015 | single | 100/105 (62) | AAP, BAP, HTG-AP | POF |
| de-Madaria, Ann Intensive Care, 2018 | Spain | prospective | for six months | multi | 39/59 (26) | AAP, BAP, PAP | severity |
| Deng, J Dig Dis, 2014 | China | retrospective | January 2008 - December 2012 | single | 1248/1894 (1146) | AAP, BAP, HTG-AP | recurrence, LOS, pseudocyst |
| Deng, Medicine, 2017 | China | prospective | September 1 2014 - November 30 2014 | single | 65/70 (54) | AAP, BAP, HTG-AP | severity |
| Dhaka, JGH Open, 2018 | India | prospective | January 2012-December 2014 | single | 411/335 (281) | AAP, BAP, PAP | necrosis |
| Dumnicka, Int J Mol Sci, 2016 | Poland | prospective | N/A | single | 58/66 (34) | AAP, BAP, HTG-AP | severity |
| Esmer, Gazi Med J, 2012 | Turkey | retrospective | January 2003 - July 2012 | single | 11/14 (0) | BAP, HTG-AP | ICU, mortality, necrosis, PUF, pseudocyst |
| Fan, Gastroenterol Res Pract, 2018 | China | retrospective | 2011, 2016 | single | 694/529 (418) | AAP, BAP, HTG-AP | severity |
| Goyal, N Am J Med Sci, 2016 | USA | retrospective | January 2009 - June 2015 | single | 177/177 (117) | AAP, HTG-AP | ICU, mortality, severity |
| Grajales-Figueroa, Gastroenterol Res Pract, 2019 | Mexico | retrospective | 1 July 1 2000-30 July 2010 | single | 96/72 (48) | AAP, BAP, HTG-AP, PAP | mortality |
| Hayashi, World J Gastroenterol, 2016 | Japan | retrospective | 12 October 2006-31 May 2007 | single | 587/36 (337) | AAP, BAP, HTG-AP | recurrence |
| He, Aging Clin Exp Res, 2020 | China | retrospective | September 2012-September 2018 | single | 198/152 (100) | AAP, BAP, HTG-AP | severity |
| Hong, BioMed Res Int, 2017 | China | N/A | January 2013 - December 2015 | single | 398/647 (406) | AAP, BAP, HTG-AP | severity |
| Huang S-W., Chin Med J (Engl), 2019 | China | retrospective | 1 January 1996-31 December 2015 | single | 5375/4241 (3137) | AAP, BAP, HTG-AP | mortality, necrosis, LOS |
| Huang Y., J Crit Care, 2019 | China | prospective | March 2013-October 2017 | multi | 1933/141 (1146) | AAP, BAP, HTG-AP | mortality |
| Huang, J Clin Apher, 2016 | China | retrospective | 1 October 2004 - 1 December 2014 | single | 13/21 (0) | BAP, HTG-AP | MOF, mortality, severity |
| Huang, Pancreas, 2014 | China | retrospective | January 1990 - December 2005 | multi | 951/1582 (417) | BAP, HTG-AP | recurrence, mortality, PUF, LOS, pseudocyst |
| Hughey, Abdom Radiol (NY), 2017 | USA | retrospective | 2004 - 2014 | single | 62/81 (61) | AAP, BAP | necrosis, fluid collection |
| Huh, J Clin Gastroenterol, 2016 | Republic of Korea | retrospective | January 2013 - June 2015 | single | 182/201 (127) | AAP, BAP, HTG-AP | mortality |
| Ikeura, Hepatobiliary Pancreat Dis Int, 2017 | Japan | N/A | January 2009 - December 2015 | single | 75/116 (78) | AAP, BAP, PAP | mortality, SIRS |
| Ivanova, Hepatobiliary Pancreat Dis Int, 2012 | Spain | prospective | 1 March 2006 - 28 February 2007 | single | 92/133 (49) | AAP, BAP, HTG-AP | recurrence, ICU, mortality |
| Jain, Clin Transl Gastroenterol, 2018 | India | prospective | January 2015-December 2016 | single | 209/157 (124) | AAP, BAP | mortality |
| Jia, Pancreas, 2015 | China | N/A | November 2011 - June 2012 | single | 62/85 (39) | AAP, BAP | severity |
| Jin, Med Sci Monit, 2017 | China | prospective | March 2013 - May 2016 | single | 558/602 (256) | AAP, BAP, HTG-AP, PAP | severity |
| Jinno, Plos One, 2019 | Japan | retrospective | 1 April 2013-31 December 2017 | multi | 209/120 (119) | AAP, BAP | mortality |
| Jones, Langenbecks Arch Surg, 2017 | UK | retrospective | 2007-2011 | N/A | 464/629 (309) | AAP, BAP, PAP | mortality |
| Kalaria, Indian J Gastroenterol, 2018 | India | prospective | 1 July 2013 - 31 December 2014 | single | 56/97 (74) | AAP, BAP | recurrence |
| Kamal, Pancreas, 2019 | Pakistan | retrospective | June 2007-June 2012 | single | 443/443 (201) | AAP, BAP, PAP | mortality, recurrence, necrosis, MOF, POF, PUF, renal failure, SIRS, pseudocyst |
| Khan, Digestion, 2013 | Finland | retrospective | N/A | single | 184/233 (166) | AAP, BAP, PAP | ICU, mortality, necrosis, LOS,  pseudocyst, |
| Kikuta, World J Gastroenterol, 2015 | Japan | N/A | 2007 | multi | 1103/1954 (1289) | AAP, BAP | mortality |
| Kim, Arch Gerontol Geriatr, 2012 | Republic of Korea | retrospective | April 2003 - March 2009 | single | 170/227 (139) | AAP, BAP | necrosis |
| Kim, Medicine (Baltimore), 2020 | Korea | retrospective | March 2010-December 2016 | single | 313/245 (209) | AAP, BAP, HTG-AP | recurrence |
| Kolber, J Clin Med, 2018 | Poland | propective | March 2014-December 2015 | single | 95/61 (65) | AAP, BAP, HTG-AP | severity |
| Koutroumpakis, Pancreatology, 2017 | USA | prospective | 2004-2008; 2009-2014 | single | 292/400 (206) | AAP, BAP, HTG-AP, PAP | MOF, mortality, severity |
| Koziel, BMC Gastroenterol, 2015 | Poland | N/A | N/A | multi | 151/221 (133) | AAP, BAP | severity |
| Kozma, J Clin Med, 2020 | Poland | prospective | January 2013-September 2014 | single | 47/47 (32) | AAP, BAP | severity |
| Kusnierz-Cabala, Pancreatology, 2015 | Poland | N/A | N/A | single | 66/88 (36) | AAP, BAP, HTG-AP | severity |
| Lankisch, Pancreatology, 2012 | Germany | prospective | January 2004- December 2006 | multi | 369/271 (N/A) | AAP, BAP | fluid collection |
| Lee, Pancreas, 2016 | Republic of Korea | prospective | March 2010 - September 2013 | single | 131/146 (92) | AAP, BAP, HTG-AP | severity |
| Lew, Pancreas, 2018 | USA | retrospective | January 2014-December 2014 | single | 175/94 (79) | AAP, BAP | recurrence, ICU, LOS |
| Li J., J Clin Lab Anal, 2020 | China | N/A | January 2016-June 2019 | single | 180/162 (112) | AAP, BAP, HTG-AP | severity |
| Li L., Dig Dis Sci, 2020 | China | retrospective | 1 January 2016-31 August 31 2017 | single | 912/617 (620) | AAP, BAP, HTG-AP | severity |
| Li Yao, Am J Med Sci, 2017 | China | N/A | February 2014 - February 2015 | single | 90/97 (50) | AAP, BAP, HTG-AP | severity |
| Li Yuanyuan, BMJ Open, 2017 | China | retrospective | 1 July 2013 - 18 August 2015 | single | 274/(359) 198 | AAP, BAP, HTG-AP | mortality, severity |
| Lin, Int J Clin Exp Med, 2016 | China | retrospective | 2008-2012 | single | 500/684 (365) | AAP, BAP, HTG-AP | mortality |
| Lin, Turk J Gastroenterol, 2019 | China | prospective | November 2016 and 2017 | single | 44/41 (22) | AAP, BAP, HTG-AP | severity |
| Lipiński, Pancreatology, 2017 | Poland | prospective | N/A | single | 104/126 (84) | AAP, BAP | severity |
| Liu J., Turk J Gastroenterol, 2017 | China | retrospective | January 2014 - July 2015 | single | 188/214 (138) | AAP, BAP, HTG-AP | POF |
| Liu T., Br J Surg, 2017 | UK | prospective | June 2010 - March 2014 | single | 168/236 (112) | AAP, BAP | severity |
| Liu, Clin Res Hepatol Gastroenterol, 2019 | China | retrospective | 1 March 2017-1 April 2018 | single | 279/240 (174) | AAP, BAP, HTG-AP | severity |
| Lu, Clin Chim Acta, 2018 | China | prospective | January 2013-January 2017 | single | 210/193 (151) | AAP, BAP, HTG-AP | severity, mortality |
| Lupia, Pancreas, 2017 | Italy | prospective | N/A | single | 29/44 (18) | AAP, BAP | severity |
| Magnusdottir, Scand J Gastroenterol, 2019 | Iceland | retrospective | 2006–2015 | multi | 1589/677 (509) | AAP, BAP | severity, mortality, recurrence |
| Maksimow, Crit Care Med, 2014 | Finland | prospective | June 2003 - February 2007 | single | 146/161 (118) | AAP, BAP | severity |
| Maleszka, Int J Mol Sci, 2017 | Poland | N/A | N/A | single | 51/66 (34) | AAP, BAP | POF, TOF, mortality, LOS, necrosis, severity |
| Mallick, JGH Open, 2018 | India | retrospective | 2010-2017 | single | 724/61 (494) | AAP, BAP, HTG-AP | severity, recurrence |
| Melitas, Pancreatology, 2019 | USA | prospective | 2015-2017 | single | 205/143 (110) | AAP, BAP, HTG-AP, PAP | recurrence |
| Mirnezami, Ann R Coll Surg Engl, 2019 | England | prospective | 1 November 2014-28 February 2015 | multi | 283/180 (125) | AAP, BAP | severity |
| Moran, United European Gastroenterol J, 2018 | Spain | prospective | June 2013–February 2015 | multi | 1655/1232 (891) | AAP, BAP | mortality, POF |
| Morton, J Clin Med, 2019 | UK | prospective | N/A | single | 15/13 (4) | AAP, BAP, PAP | severity |
| Mounzer, Gastroenterology, 2012 | USA | prospective | July 2003 - August 2010; June 2005 - December 2007 | multi | 332/653 (217) | AAP, BAP | POF |
| Nawacki, Acta Gastroenterol Belg, 2019 | Poland | prospective | N/A | single | 72/67 (38) | AAP, BAP | severity |
| Nebiker, Pancreas, 2018 | Switzerland | prospective | April 2011 to January 2015 | single | 103/142 (99) | AAP, BAP | severity |
| Nukarinen, Plos One, 2016 | Finland | retrospective | March 2011 - August 2014 | single | 148/176 (122) | AAP, BAP | severity |
| Párniczky, Plos One, 2016* | Hungary | prospective | 1 January 2013 - 1 January 2015 | multi | 397/600 (335) | AAP, BAP, HTG-AP, PAP | severity |
| Peng, Am J Emerg Med, 2017 | China | retrospective | January 2014 - May 2015 | single | 125/128 (71) | AAP, BAP, HTG-AP | POF |
| Popov, J Clin Monit Comput, 2017 | Russia | N/A | September 2009 - July 2012 | single | 49/49 (30) | AAP, BAP | severity |
| Pulkkinen, Pancreas, 2014 | Finland | retrospective | 2008-2010 | single | 388/461 (299) | AAP, BAP | mortality |
| Quero, Scand J Gastroenterol, 2019 | Italy | retrospective | 1 January 2008-31 December 2017 | single | 884/587 (498) | AAP, BAP | mortality, ICU, LOS |
| Radovanović-Dinić, Turk J Gastroenterol, 2018 | Serbia | prospective | January 2012-August 2016 | single | 92/80 (45) | AAP, BAP | severity |
| Rainio, Pancreas, 2019 | Finland | N/A | 2005–2012 | single | 239/216 (176) | AAP, BAP | severity |
| Reid, Ann Med Surg, 2017 | Jamaica | retrospective | 1 January 2006 - 31 December 2012 | single | 78/91 (21) | AAP, BAP, HTG-AP, PAP | severity |
| Roberts, BMC Gastroenterol, 2014 | UK | retrospective | January 1 1999 - December 31 2010 | multi | 6230/10589 (5585) | AAP, BAP | mortality |
| Samanta, JGH Open, 2019 | India | retrospective | January 2010, June 2018 | single | 759/614 (442) | AAP, BAP | mortality, recurrence, ICU, MOF, PUF, renal failure, LOS |
| Seok, Korean J Gastroenterol, 2019 | Korea | N/A | June 2016-August 2018 | single | 49/38 (18) | AAP, BAP | LOS |
| Skouras, HPB, 2014 | UK | retrospective cohort (prospectively maintained database) | January 2000-December 2004 | single | 560/694 (355) | AAP, BAP | MOF, mortality |
| Skouras, HPB, 2016 | UK | prospective | September 2013 - December 2013 | single | 29/41 (21) | AAP, BAP | severity |
| Sternby, United European Gastroenterol J, 2019 | Sweden, Finland, Spain, Estonia, Lithuania, Bulgaria, Germany | retrospective | January 2012-January 2013, in the two German centres: January 2010-January 2016 | multi | 454/324 (264) | AAP, BAP | severity |
| Sue, Pancreas, 2017 | USA | retrospective | 1 January 2006-31 December 2013 | multi | 2519/1179 (1274) | AAP, BAP | TOF |
| Sun, BMC Pregnancy Childbirth, 2015 | China | retrospective | January 2006 - December 2013 | single | 16/17 (0) | AAP, BAP, HTG-AP | severity |
| Takuma, Adv Med Sci, 2012 | Japan | N/A | January 1975 - December 2010 | single | 202/381 (238) | AAP, BAP, HTG-AP | recurrence |
| Verdonk, Eur J Gastroenterol Hepatol, 2018 | The Netherlands | post-hoc analysis of a previously established multicentre cohort | N/A | multi | 285/207 (159) | AAP, BAP | severity, necrosis, POF, PUF, renal failure, TOF |
| Vujasinovic, World J Gastroenterol, 2014 | Slovenia | N/A | N/A | multi | 18/100 (65) | AAP, BAP | recurrence, severity |
| Wan, BMC Gastroenterol, 2019 | China | retrospective | 1 January 2014-31 December 2017 | single | 2478/2308 (1915) | AAP, BAP, HTG-AP | severity |
| Wan, Lipids Health Dis, 2017 | China | retrospective | 1 January 2005 - 31 December 2013 | single | 1130/1539 (848) | AAP, BAP, HTG-AP | severity |
| Wang, Curr Med Res Opin, 2018 | China | retrospective | January 2013 - December 2015 | single | 322/480 (278) | AAP, BAP, HTG-AP, PAP | severity |
| Weitz, JOP, 2015 | Germany | retrospective | January 2008-December 2011 | single | 268/391 (155) | AAP, BAP | recurrence, POF, LOS, mortality, necrosis |
| Wu, Dig Dis Sci, 2018 | China | prospective | June 2015-June 2017 | single | 74/36 (47) | AAP, BAP, HTG-AP | severity |
| Yadav, Am J Gastroenterol, 2012 | USA | N/A | 1996 - 2005 | single | 3474/7456 (3318) | AAP, BAP | mortality, LOS |
| Yadav, Pancreas, 2014 | USA | N/A | 1996-2005 | multi | 6010/2870 (2594) | AAP, BAP | recurrence |
| Yang, J Dig Dis, 2018 | China | retrospective | January 2012-August 2017 | single | 172/111 (105) | AAP, BAP, HTG-AP | severity |
| Youn, Korean J Gastroenterol, 2017 | Republic of Korea | retrospective | January 2003 - January 2013 | single | 905/1110 (767) | AAP, BAP | recurrence, mortality |
| Yu, Dig Dis Sci, 2020 | China | N/A | June 2018-April 2019 | single | 60/50 (31) | AAP, BAP, HTG-AP | severity |
| Zádori, United European Gastroenterol J, 2020 | intrenational (13 countries) | prospective | 2012-2019 | multi | 2400/1495 (N/A) | AAP, BAP, HTG-AP | recurrence |
| Zhang G-Q., Med Sci Monit, 2019 | China | retrospective | March 2012-March 2017 | single | 334/313 (220) | AAP, BAP, HTG-AP | severity |
| Zhang L., J Clin Lab Anal, 2020 | China | N/A | January 2015-December 2016 | single | 164/140 (88) | AAP, BAP, HTG-AP | severity |
| Zhang Q., J Clin Lab Anal, 2020 | China | retrospective | January 2016-December 2018 | single | 225/200 (140) | AAP, BAP, HTG-AP | severity |
| Zhang R., J Immunol, 2019 | China | prospective | 2016-2017 | single | 50/45 (39) | AAP, BAP, HTG-AP | severity |
| Zhang, Dig Dis Sci, 2016 | China | prospective | March 2014 - March 2015 | single | 102/120 (74) | AAP, BAP, HTG-AP | severity |
| Zhang T., Shock, 2018 | China | retrospective | January 2009 - October 2015 | single | 446/545 (304) | AAP, BAP, HTG-AP | severity |
| Zhang X., Tohoku J Exp Med, 2018 | China | N/A | N/A | single | 15/13 (9) | BAP, HTG-AP | severity, mortality, PUF |
| Zhao, Am J Med Sci, 2019 | China | retrospective | January 2015-June 2017 | N/A | 164/140 (88) | AAP, BAP, HTG-AP | severity |
| Zhao, J Dig Dis, 2016 | China | prospective | June 2012 - September 2014 | single | 66/74 (47) | BAP, HTG-AP, PAP | severity |
| Zhou, Medicine (Baltimore), 2019 | China | retrospective | January 2014-December 2017 | single | 406/356 (242) | AAP, BAP, HTG-AP | severity, mortality |
| Zhu, Pancreas, 2017 | China | N/A | January 2005 - December 2012 | single | 2529/3260 (1721) | AAP, BAP, HTG-AP | mortality |

*The authors had access to the raw data in Párniczky et al. (2016)

**Supplementary Table S4.** Risk of bias of the analysed articles. Abbreviation: PF: prognostic factor

| **Author** | **1. Study participation** | **2. Study Attrition** | **3. PF Measurement** | **4. Outcome Measurement** | **5. Study Confounding** | **6. Statistical Analysis and Reporting** | **All domains** |
| --- | --- | --- | --- | --- | --- | --- | --- |
| **Avanesov, 2016** |  |  |  |  |  |  |  |
| **Avanesov, 2018** |  |  |  |  |  |  |  |
| **Badhal, 2012** |  |  |  |  |  |  |  |
| **Berger, 2020** |  |  |  |  |  |  |  |
| **Bertilsson, 2015** |  |  |  |  |  |  |  |
| **Bertilsson, 2016** |  |  |  |  |  |  |  |
| **Bishu, 2018** |  |  |  |  |  |  |  |
| **Bogdan, 2012** |  |  |  |  |  |  |  |
| **Bosques-Padilla, 2015** |  |  |  |  |  |  |  |
| **Buxbaum, 2018** |  |  |  |  |  |  |  |
| **Castoldi, 2013** |  |  |  |  |  |  |  |
| **Cavestro, 2015** |  |  |  |  |  |  |  |
| **Ćeranić, 2019** |  |  |  |  |  |  |  |
| **Chacón-Portillo, 2017** |  |  |  |  |  |  |  |
| **Chen, 2013** |  |  |  |  |  |  |  |
| **Chen, 2017** |  |  |  |  |  |  |  |
| **Chen, 2019** |  |  |  |  |  |  |  |
| **Cho, 2015** |  |  |  |  |  |  |  |
| **Cho, 2018** |  |  |  |  |  |  |  |
| **Cho, 2020** |  |  |  |  |  |  |  |
| **Choi, 2014** |  |  |  |  |  |  |  |
| **Cui, 2014** |  |  |  |  |  |  |  |
| **Cui, 2017** |  |  |  |  |  |  |  |
| **de-Madaria, 2018** |  |  |  |  |  |  |  |
| **Deng, 2014** |  |  |  |  |  |  |  |
| **Deng, 2017** |  |  |  |  |  |  |  |
| **Dhaka, 2018** |  |  |  |  |  |  |  |
| **Dumnicka, 2016** |  |  |  |  |  |  |  |
| **Esmer, 2012** |  |  |  |  |  |  |  |
| **Fan, 2018** |  |  |  |  |  |  |  |
| **Goyal, 2016** |  |  |  |  |  |  |  |
| **Grajales-Figueroa, 2019** |  |  |  |  |  |  |  |
| **Hayashi, 2016** |  |  |  |  |  |  |  |
| **He, 2020** |  |  |  |  |  |  |  |
| **Hong, 2017** |  |  |  |  |  |  |  |
| **Huang, 2014** |  |  |  |  |  |  |  |
| **Huang, 2016** |  |  |  |  |  |  |  |
| **Huang Y., 2019** |  |  |  |  |  |  |  |
| **Huang S-W., 2019** |  |  |  |  |  |  |  |
| **Hughey, 2017** |  |  |  |  |  |  |  |
| **Huh, 2016** |  |  |  |  |  |  |  |
| **Ikeura, 2017** |  |  |  |  |  |  |  |
| **Ivanova, 2012** |  |  |  |  |  |  |  |
| **Jain, 2018** |  |  |  |  |  |  |  |
| **Jia, 2015** |  |  |  |  |  |  |  |
| **Jin, 2017** |  |  |  |  |  |  |  |
| **Jinno, 2019** |  |  |  |  |  |  |  |
| **Jones, 2017** |  |  |  |  |  |  |  |
| **Kalaria, 2018** |  |  |  |  |  |  |  |
| **Kamal, 2019** |  |  |  |  |  |  |  |
| **Khan, 2013** |  |  |  |  |  |  |  |
| **Kikuta, 2015** |  |  |  |  |  |  |  |
| **Kim, 2012** |  |  |  |  |  |  |  |
| **Kim, 2020** |  |  |  |  |  |  |  |
| **Kolber, 2018** |  |  |  |  |  |  |  |
| **Koutroumpakis, 2017** |  |  |  |  |  |  |  |
| **Koziel, 2015** |  |  |  |  |  |  |  |
| **Kozma, 2020** |  |  |  |  |  |  |  |
| **Kusnierz-Cabala, 2015** |  |  |  |  |  |  |  |
| **Lankish, 2012** |  |  |  |  |  |  |  |
| **Lee, 2016** |  |  |  |  |  |  |  |
| **Lew, 2018** |  |  |  |  |  |  |  |
| **Li J., 2020** |  |  |  |  |  |  |  |
| **Li L., 2020** |  |  |  |  |  |  |  |
| **Li Yao, 2017** |  |  |  |  |  |  |  |
| **Li Yuanyuan, 2017** |  |  |  |  |  |  |  |
| **Lin, 2016** |  |  |  |  |  |  |  |
| **Lin, 2019** |  |  |  |  |  |  |  |
| **Lipiński, 2017** |  |  |  |  |  |  |  |
| **Liu J., 2017** |  |  |  |  |  |  |  |
| **Liu T., 2017** |  |  |  |  |  |  |  |
| **Liu, 2019** |  |  |  |  |  |  |  |
| **Lu, 2018** |  |  |  |  |  |  |  |
| **Lupia, 2017** |  |  |  |  |  |  |  |
| **Magnusdottir, 2019** |  |  |  |  |  |  |  |
| **Maksimow, 2014** |  |  |  |  |  |  |  |
| **Maleszka, 2017** |  |  |  |  |  |  |  |
| **Mallick, 2018** |  |  |  |  |  |  |  |
| **Melitas, 2019** |  |  |  |  |  |  |  |
| **Mirnezami, 2019** |  |  |  |  |  |  |  |
| **Moran, 2018** |  |  |  |  |  |  |  |
| **Morton, 2019** |  |  |  |  |  |  |  |
| **Mounzer, 2012** |  |  |  |  |  |  |  |
| **Nawacki, 2019** |  |  |  |  |  |  |  |
| **Nebiker, 2018** |  |  |  |  |  |  |  |
| **Nukarinen, 2016** |  |  |  |  |  |  |  |
| **Párniczky, 2016*** |  |  |  |  |  |  |  |
| **Peng, 2017** |  |  |  |  |  |  |  |
| **Popov, 2017** |  |  |  |  |  |  |  |
| **Pulkkinen, 2014** |  |  |  |  |  |  |  |
| **Quero, 2019** |  |  |  |  |  |  |  |
| **Radovanović-Dinić, 2018** |  |  |  |  |  |  |  |
| **Rainio, 2019** |  |  |  |  |  |  |  |
| **Reid, 2017** |  |  |  |  |  |  |  |
| **Roberts, 2014** |  |  |  |  |  |  |  |
| **Samanta, 2019** |  |  |  |  |  |  |  |
| **Seok, 2019** |  |  |  |  |  |  |  |
| **Skouras, 2014** |  |  |  |  |  |  |  |
| **Skouras, 2016** |  |  |  |  |  |  |  |
| **Sternby, 2019** |  |  |  |  |  |  |  |
| **Sue, 2017** |  |  |  |  |  |  |  |
| **Sun, 2015** |  |  |  |  |  |  |  |
| **Takuma, 2012** |  |  |  |  |  |  |  |
| **Verdonk, 2018** |  |  |  |  |  |  |  |
| **Vujasinovic, 2014** |  |  |  |  |  |  |  |
| **Wan, 2017** |  |  |  |  |  |  |  |
| **Wan, 2019** |  |  |  |  |  |  |  |
| **Wang, 2018** |  |  |  |  |  |  |  |
| **Weitz, 2015** |  |  |  |  |  |  |  |
| **Wu, 2018** |  |  |  |  |  |  |  |
| **Yadav, 2012** |  |  |  |  |  |  |  |
| **Yadav, 2014** |  |  |  |  |  |  |  |
| **Yang, 2018** |  |  |  |  |  |  |  |
| **Youn, 2017** |  |  |  |  |  |  |  |
| **Yu, 2020** |  |  |  |  |  |  |  |
| **Zádori, 2020** |  |  |  |  |  |  |  |
| **Zhang G-Q., 2019** |  |  |  |  |  |  |  |
| **Zhang L., 2020** |  |  |  |  |  |  |  |
| **Zhang Q., 2020** |  |  |  |  |  |  |  |
| **Zhang R., 2019** |  |  |  |  |  |  |  |
| **Zhang T., 2018** |  |  |  |  |  |  |  |
| **Zhang X., 2018** |  |  |  |  |  |  |  |
| **Zhang, 2016** |  |  |  |  |  |  |  |
| **Zhao, 2016** |  |  |  |  |  |  |  |
| **Zhao, 2019** |  |  |  |  |  |  |  |
| **Zhou, 2019** |  |  |  |  |  |  |  |
| **Zhu, 2017** |  |  |  |  |  |  |  |

**Supplementary Figure S1.** The quality of the articles in our meta-analysis was assessed by the Quality In Prognostic Stuidies (QUIPS) tool. The following figure gives an overview per domain.

**Supplementary Figure S2.** Forest plot showing the comparison of disease severity in PAP and BAP, p=0.223. Filled rhombuses represent the OR derived from the articles analysed. Horizontal bars represent CI. Empty rhombus shows the overall OR (OR is the middle of the rhombus and 95% CIs are the edges) for non-mild disease.


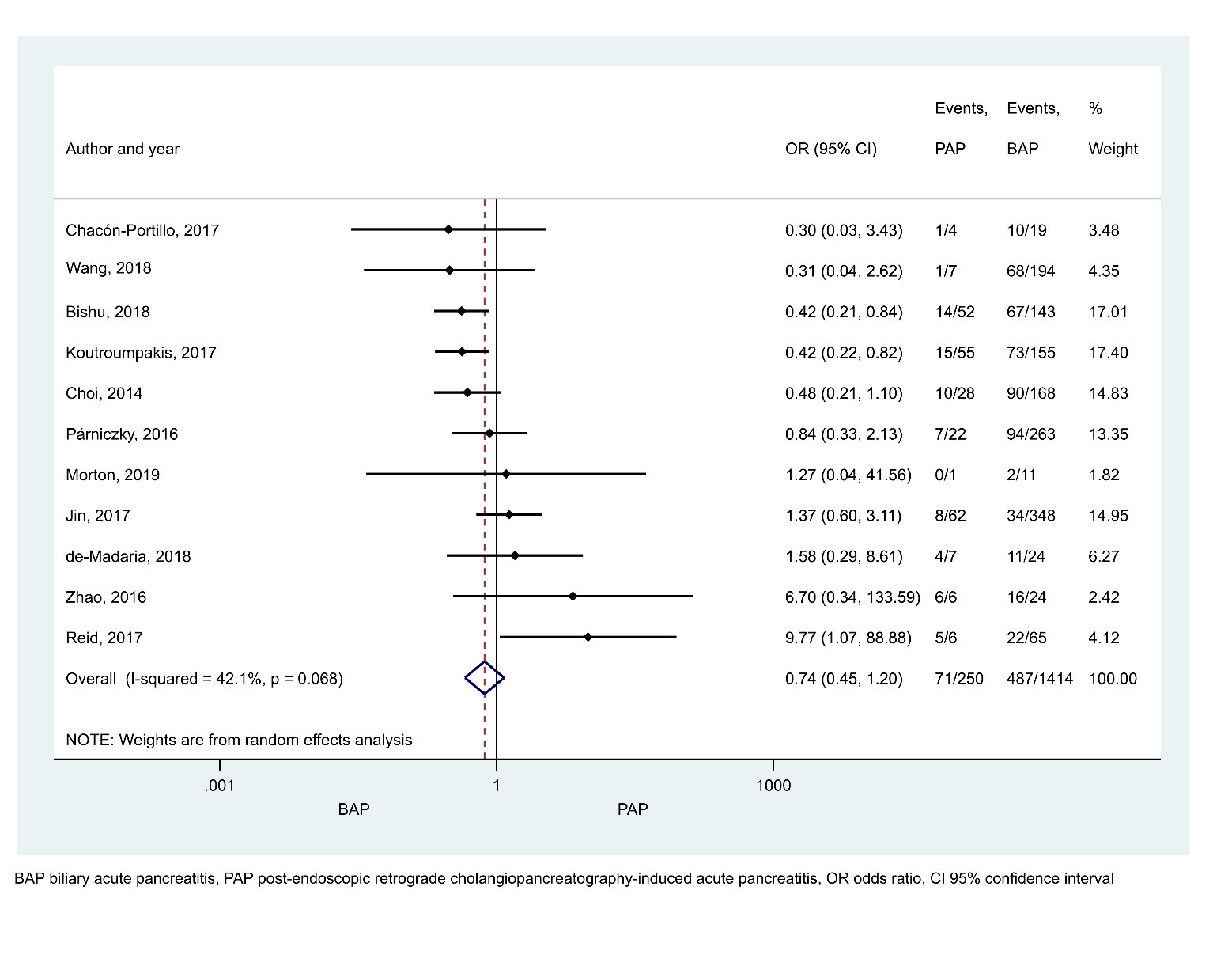


**Supplementary Figure S3.** Funnel plots of severity related to Figure 2, 4 and Supplementary Figure S1. (a) AAP vs. HTG-AP, p=0.648; (b) BAP vs. HTG-AP, p=0.787; (c) AAP vs. BAP, p=0.667; (D) BAP vs. PAP, p=0.306. The two oblique lines mark the pseudo-95% confidence limits.


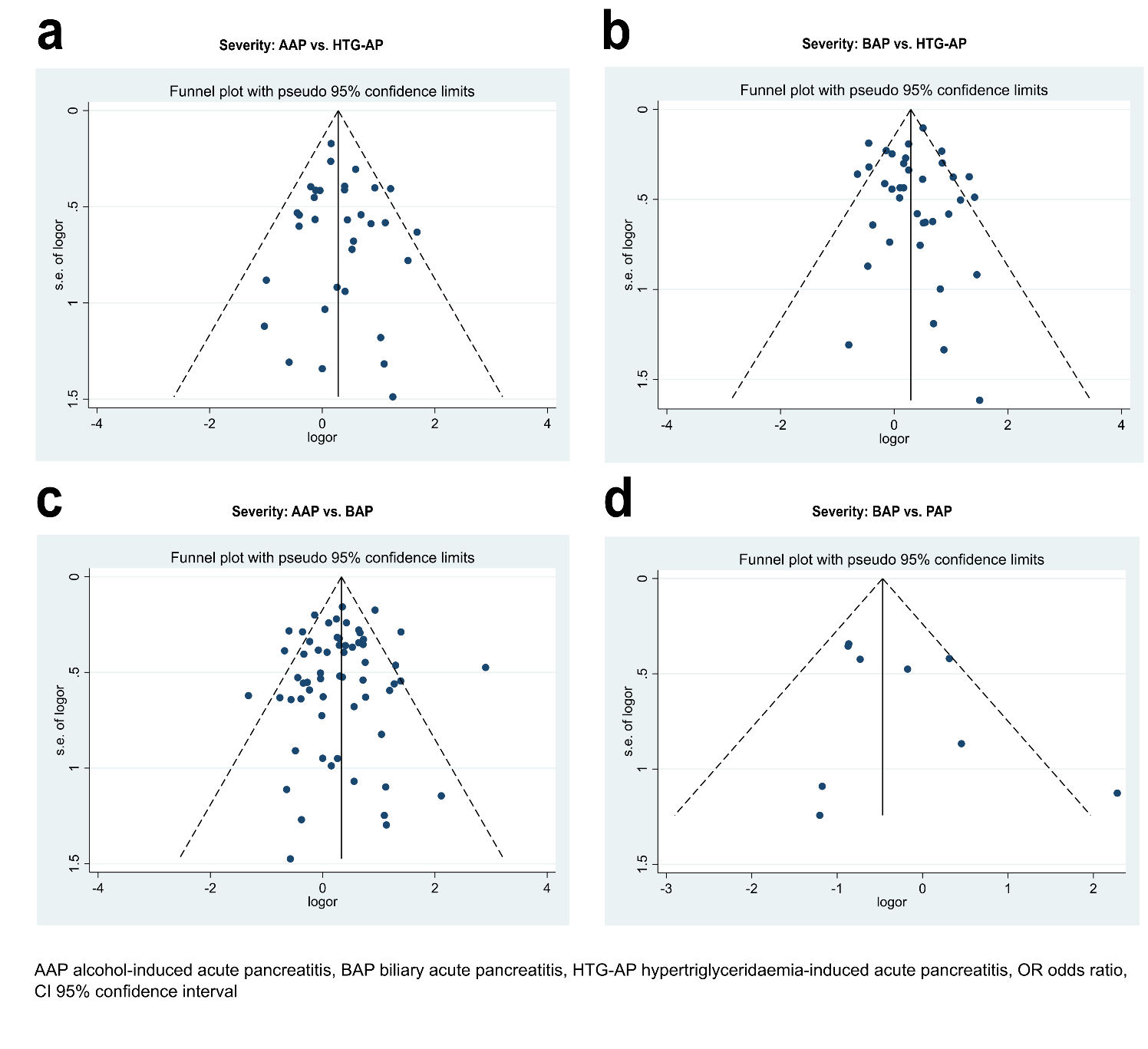


**Supplementary Figure S4.** Forest plot showing the effect of different disease aetiologies on POF. The effects of (a) HTG-AP and AAP, p=0.204; (b) HTG-AP and BAP, p=0.612. Filled rhombuses represent the ORs derived from the articles analysed. Horizontal bars represent CI. Empty rhombus shows the overall OR (the middle of the rhombus, CIs are the edges).


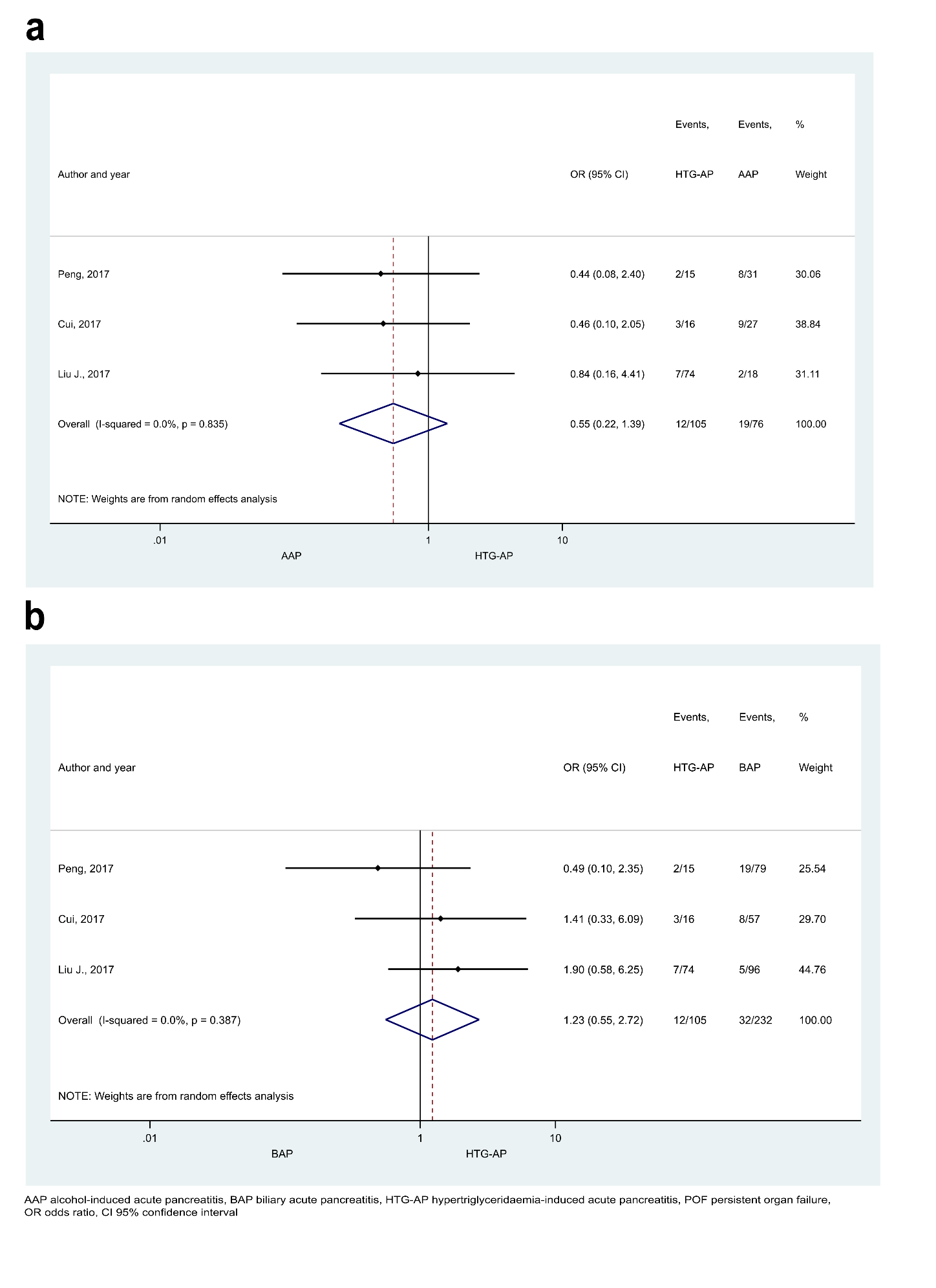


**Supplementary Figure S5.** Funnel plots related to Figures 5b, 7a, Supplementary Figures S10a, S12a, comparing AAP to BAP. (a) POF, p=0.644; (b) recurrence rate, p=0.234; (c) LOS, p=0.720, (d) necrosis, p=0.986. The two oblique lines mark the pseudo-95% confidence limits.


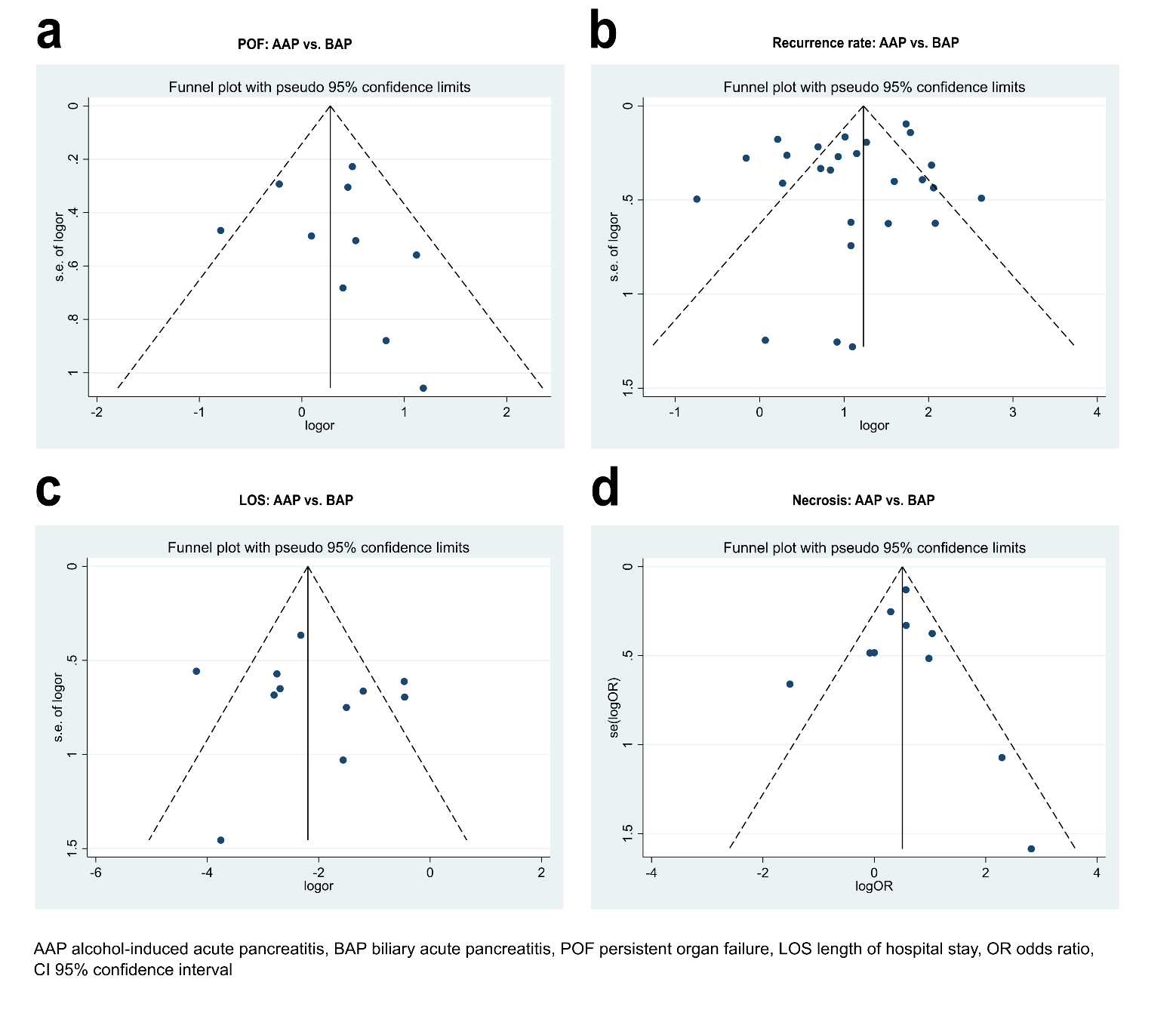


**Supplementary Figure S6.** Forest plot showing the effect of AAP and BAP on (a) TOF, p=0.656; (b) renal failure, p=0.163. Filled rhombuses represent the ORs derived from the articles analysed. Horizontal bars represent CI. Empty rhombus shows the overall OR (the middle of the rhombus, CIs are the edges).


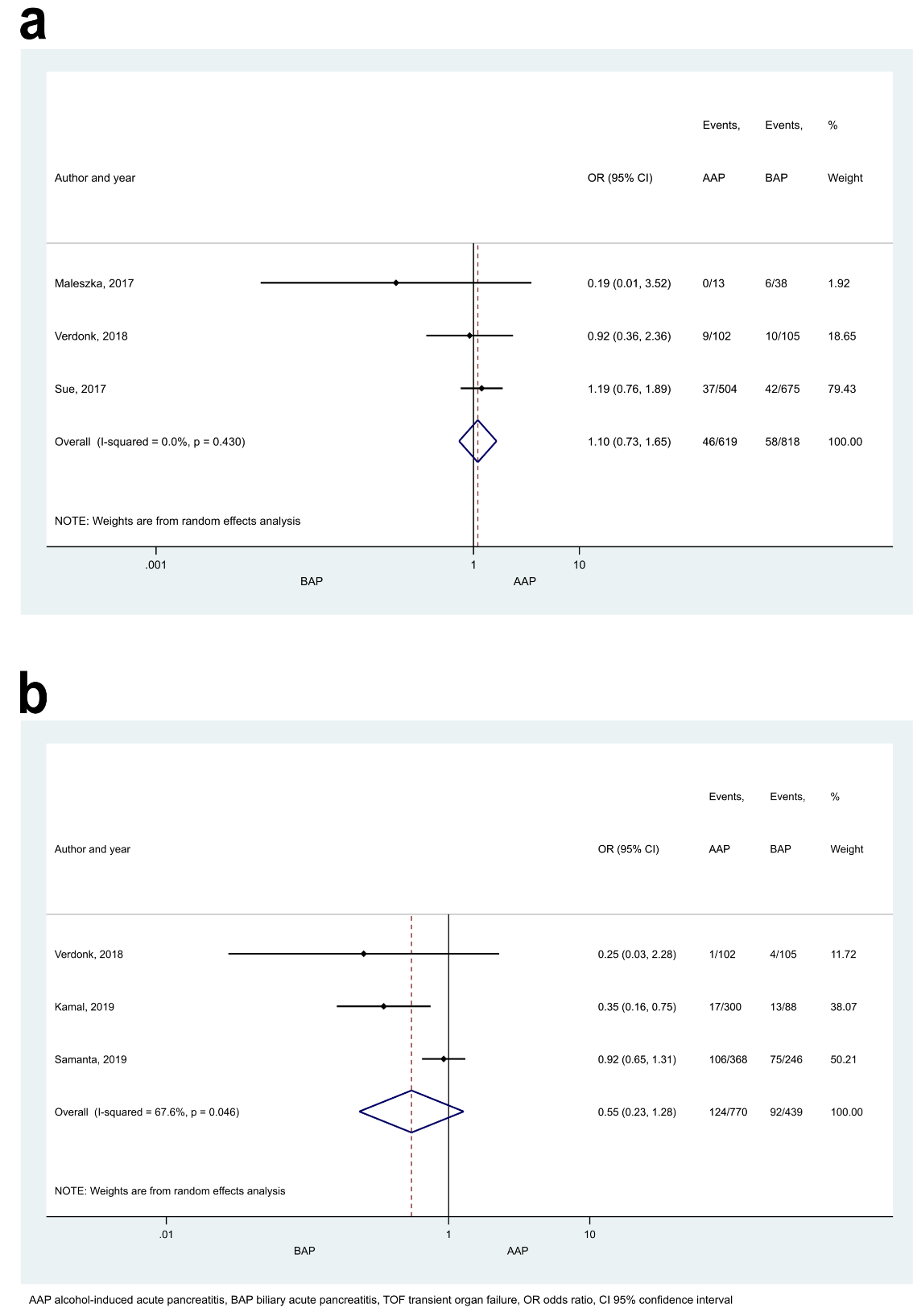


**Supplementary Figure S7.** Forest plot showing the effect of different disease aetiologies on PUF. The effects of (a) HTG-AP and BAP, p=0.0036; (b) AAP and BAP, p=0.371. Filled rhombuses represent the ORs derived from the articles analysed. Horizontal bars represent CI. Empty rhombus shows the overall OR (the middle of the rhombus, CIs are the edges).


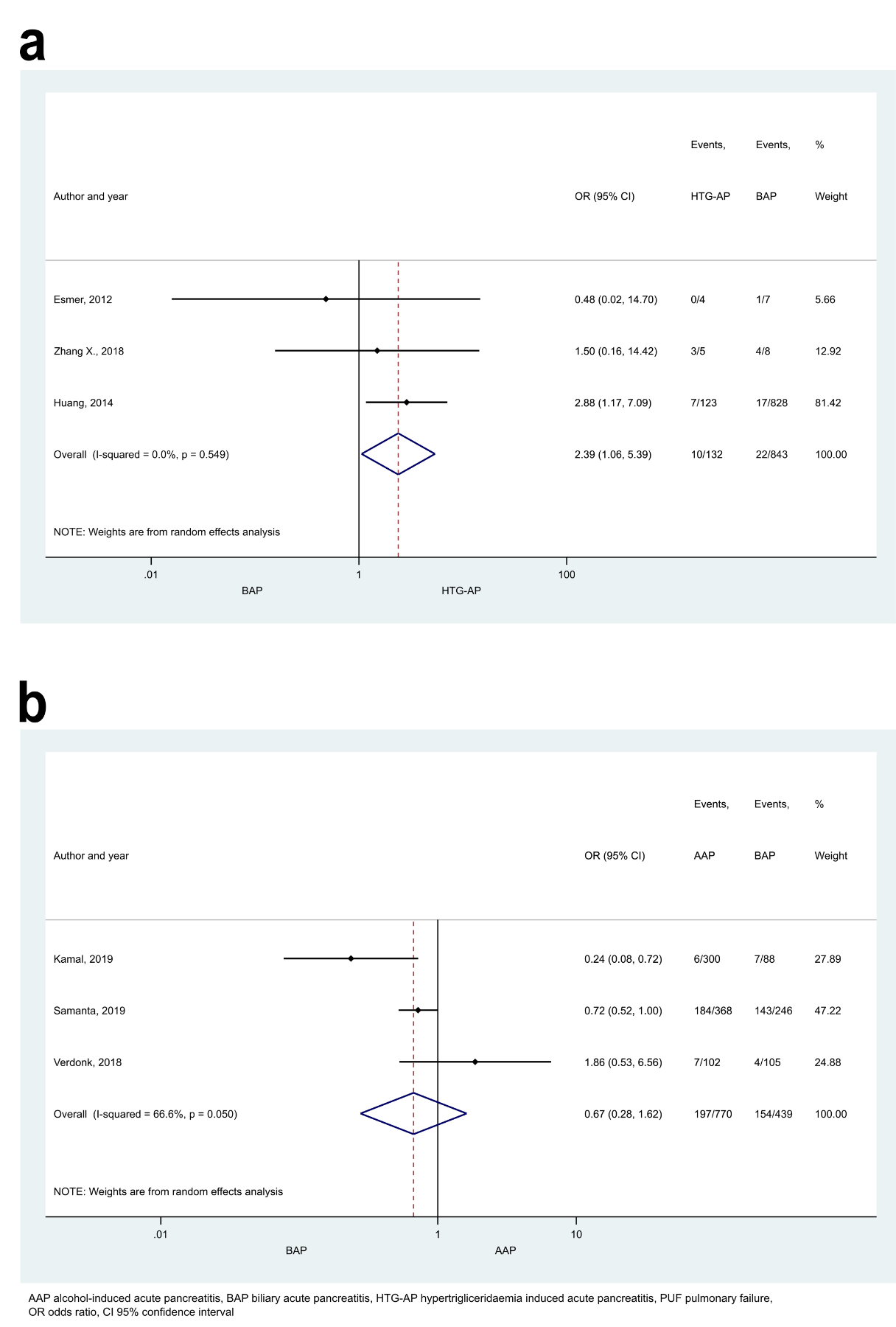


**Supplementary Figure S8.** Forest plot showing the effect of AAP and BAP on ICU admission, p=0.742. Filled rhombuses represent the ORs derived from the articles analysed. Horizontal bars represent CI. Empty rhombus shows the overall OR (the middle of the rhombus, CIs are the edges).


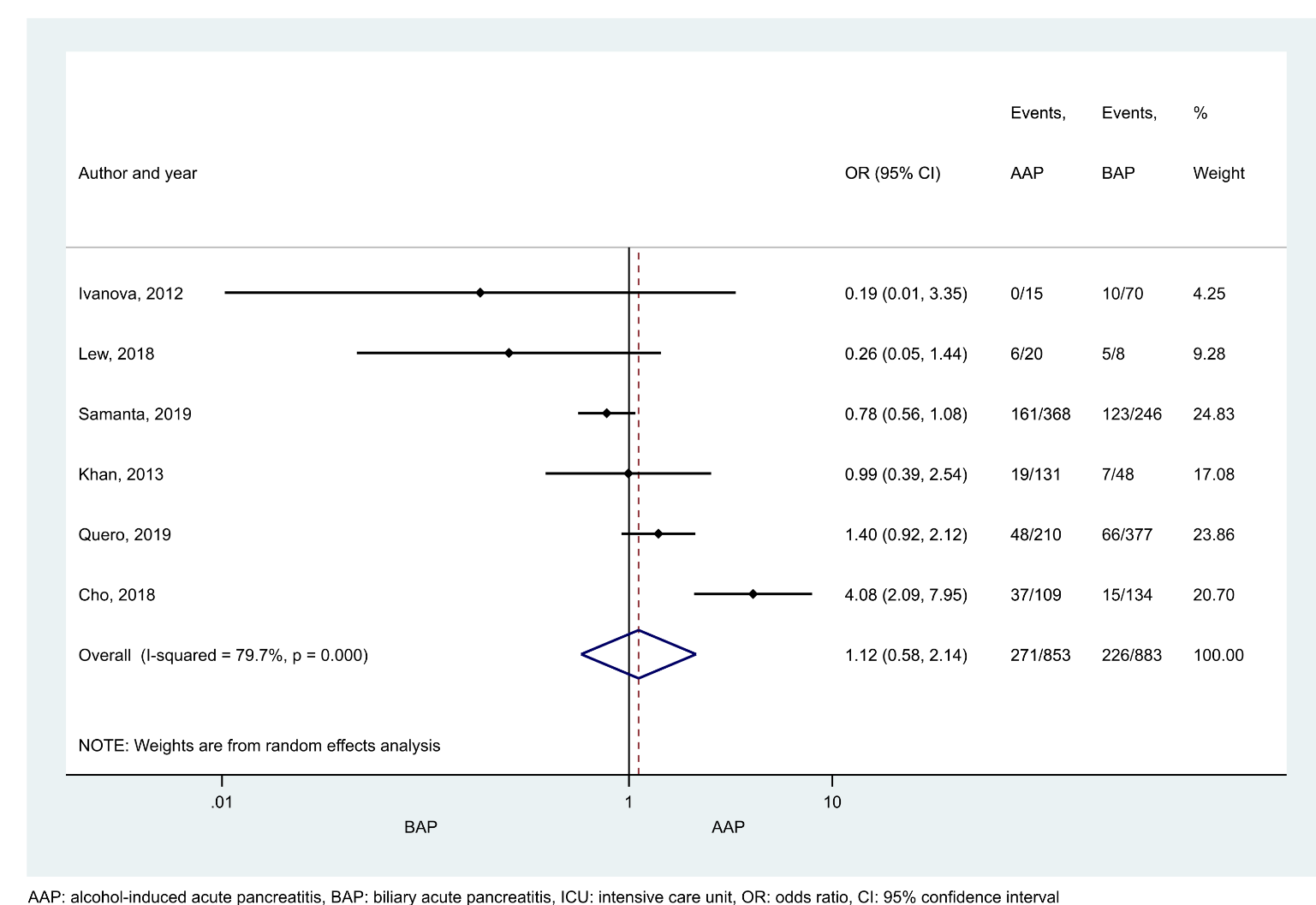


**Supplementary Figure S9.** Forest plot showing the effect of different disease aetiologies on SIRS. The effects of (a) AAP and PAP, p=0.006; (b) AAP and BAP, p=0.733; (c) BAP and PAP, p=0.109. Filled rhombuses represent the ORs derived from the articles analysed. Horizontal bars represent CI. Empty rhombus shows the overall OR (the middle of the rhombus, CIs are the edges).


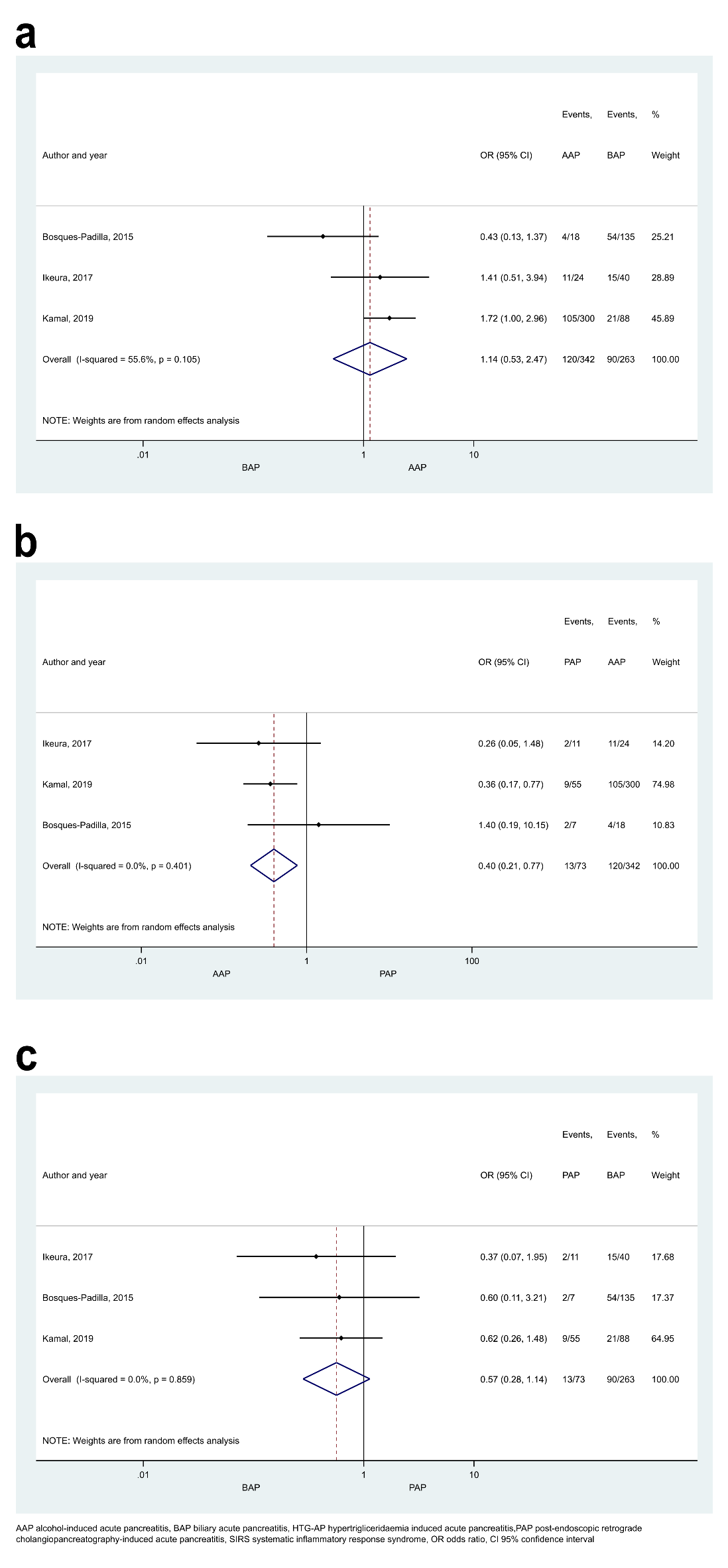


**Supplementary Figure S10.** Forest plot showing the effect of different disease aetiologies on recurrence rate. The effects of (a) AAP and BAP, p<0.001; (b) HTG-AP and BAP, p<0.001. Filled rhombuses represent the ORs derived from the articles analysed. Horizontal bars represent CI. Empty rhombus shows the overall OR (the middle of the rhombus, CIs are the edges).


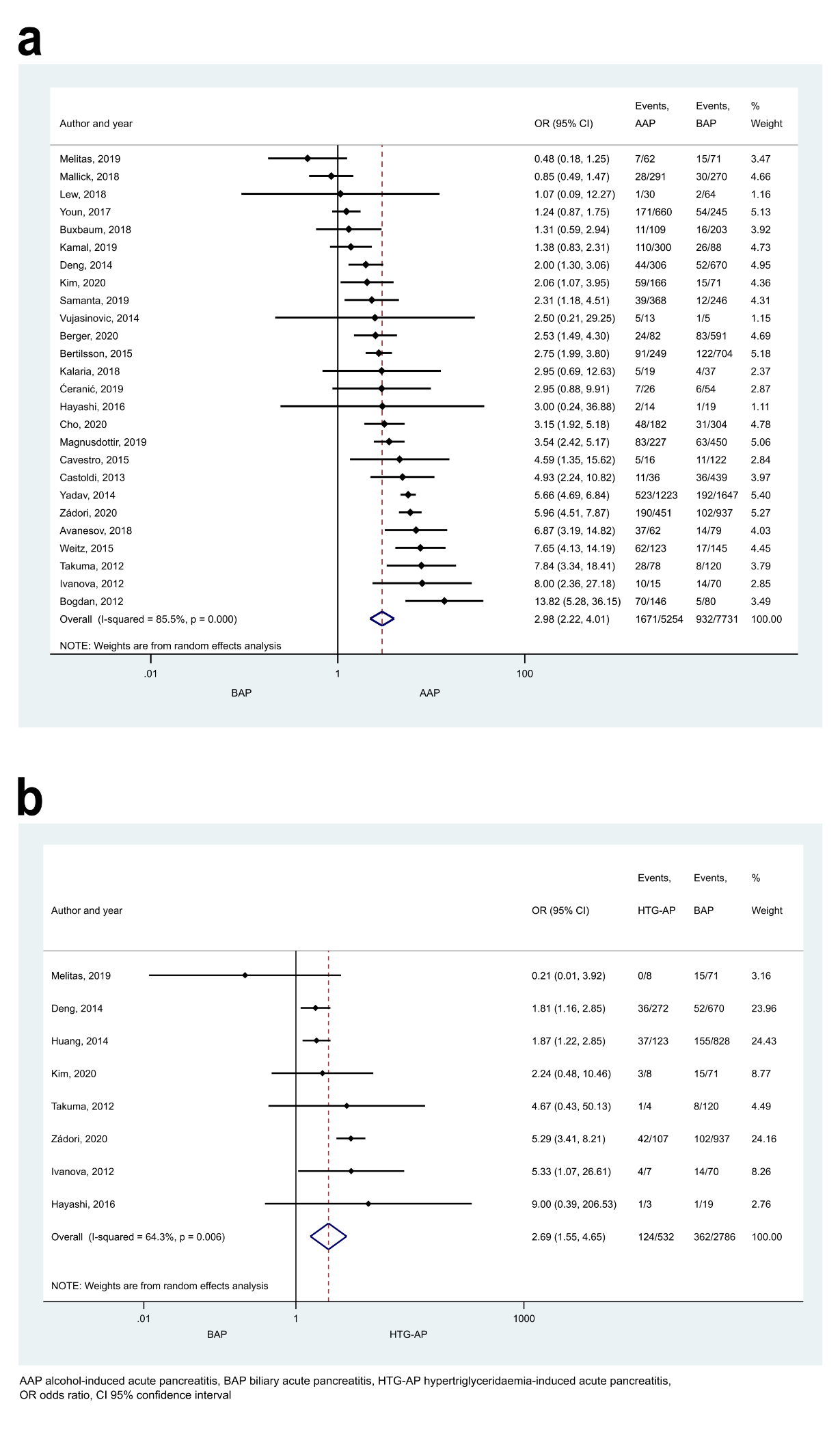


**Supplementary Figure S11.** Forest plot showing the effect of different disease aetiologies on recurrence rate. The effects of (a) HTG-AP and AAP, p=0.477; (b) AAP and PAP, p=0.572; (c) BAP and PAP, p=0.900. Filled rhombuses represent the ORs derived from the articles analysed. Horizontal bars represent CI. Empty rhombus shows the overall OR (the middle of the rhombus, CIs are the edges).

**
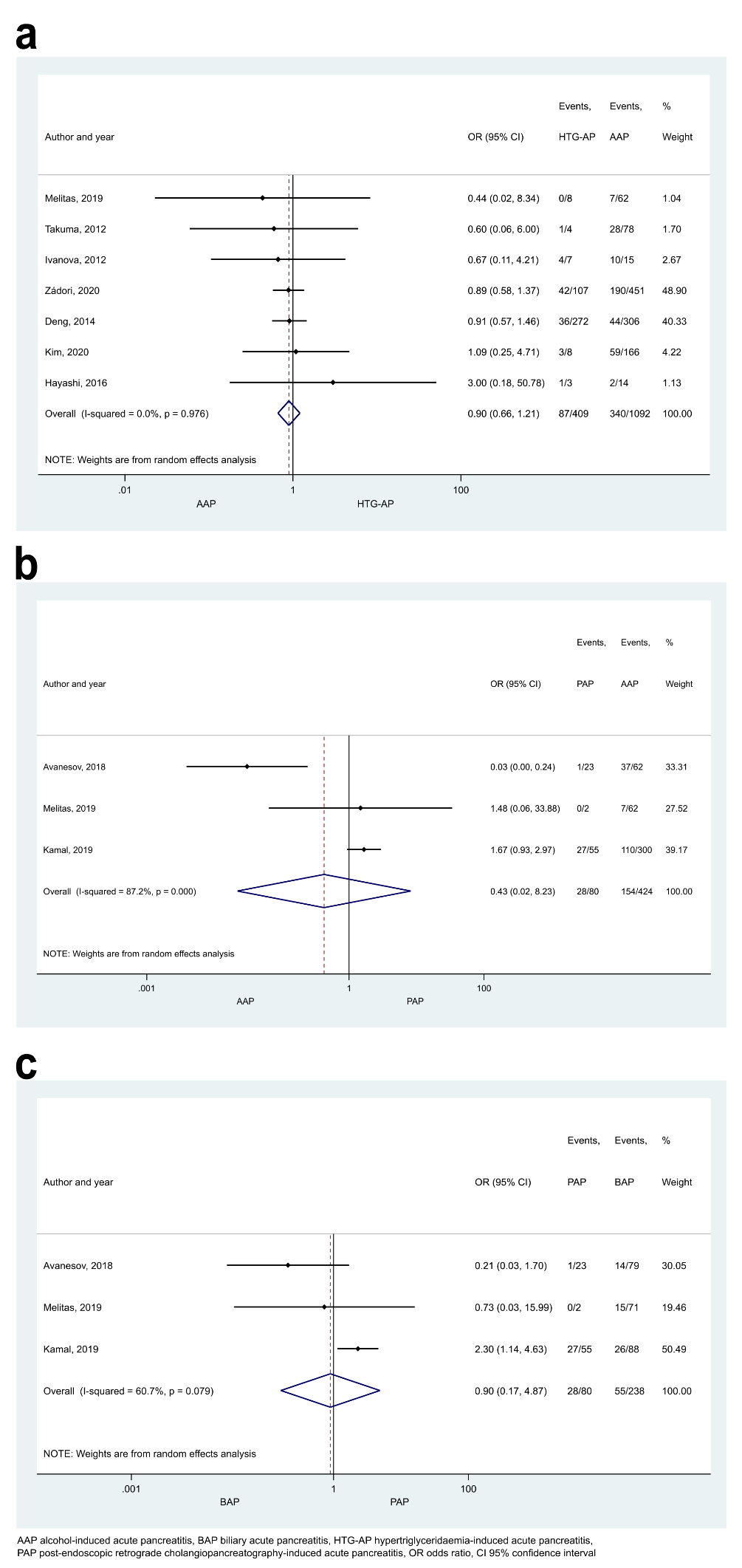
**

**Supplementary Figure S12.** Forest plot showing the effect of different disease aetiologies on LOS. The effects of (a) AAP and BAP, p=0.334; (b) HTG-AP and BAP, p=0.324. Filled rhombuses represent the ORs derived from the articles analysed. Horizontal bars represent CI. Empty rhombus shows the overall OR (the middle of the rhombus, CIs are the edges).


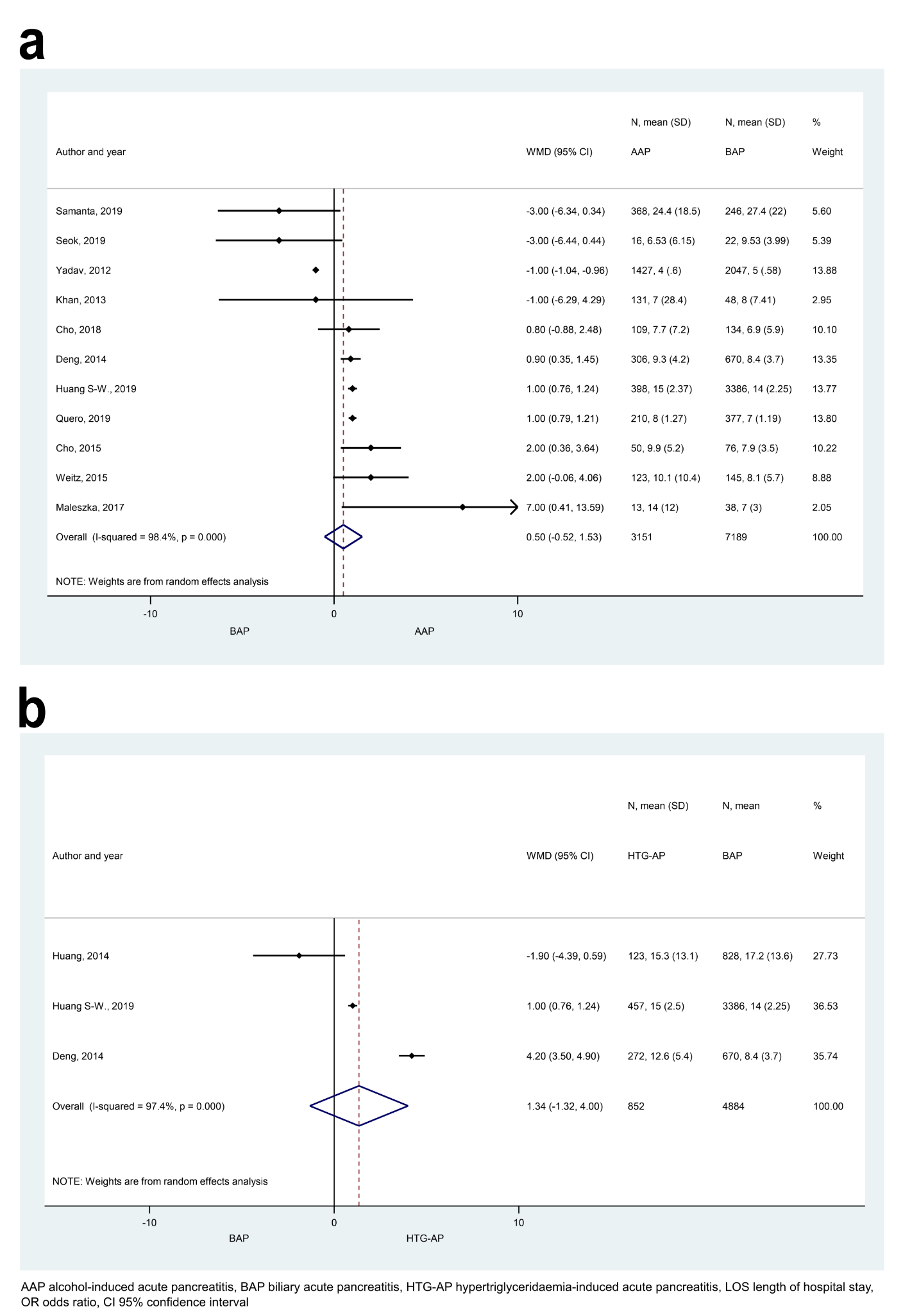


**Supplementary Figure S13.** Forest plot showing the effect of different disease aetiologies on mortality. The effects of (a) AAP and BAP, p=0.175; (b) HTG-AP and BAP, p=0.074. Filled rhombuses represent the ORs derived from the articles analysed. Horizontal bars represent CI. Empty rhombus shows the overall OR (the middle of the rhombus, CIs are the edges).


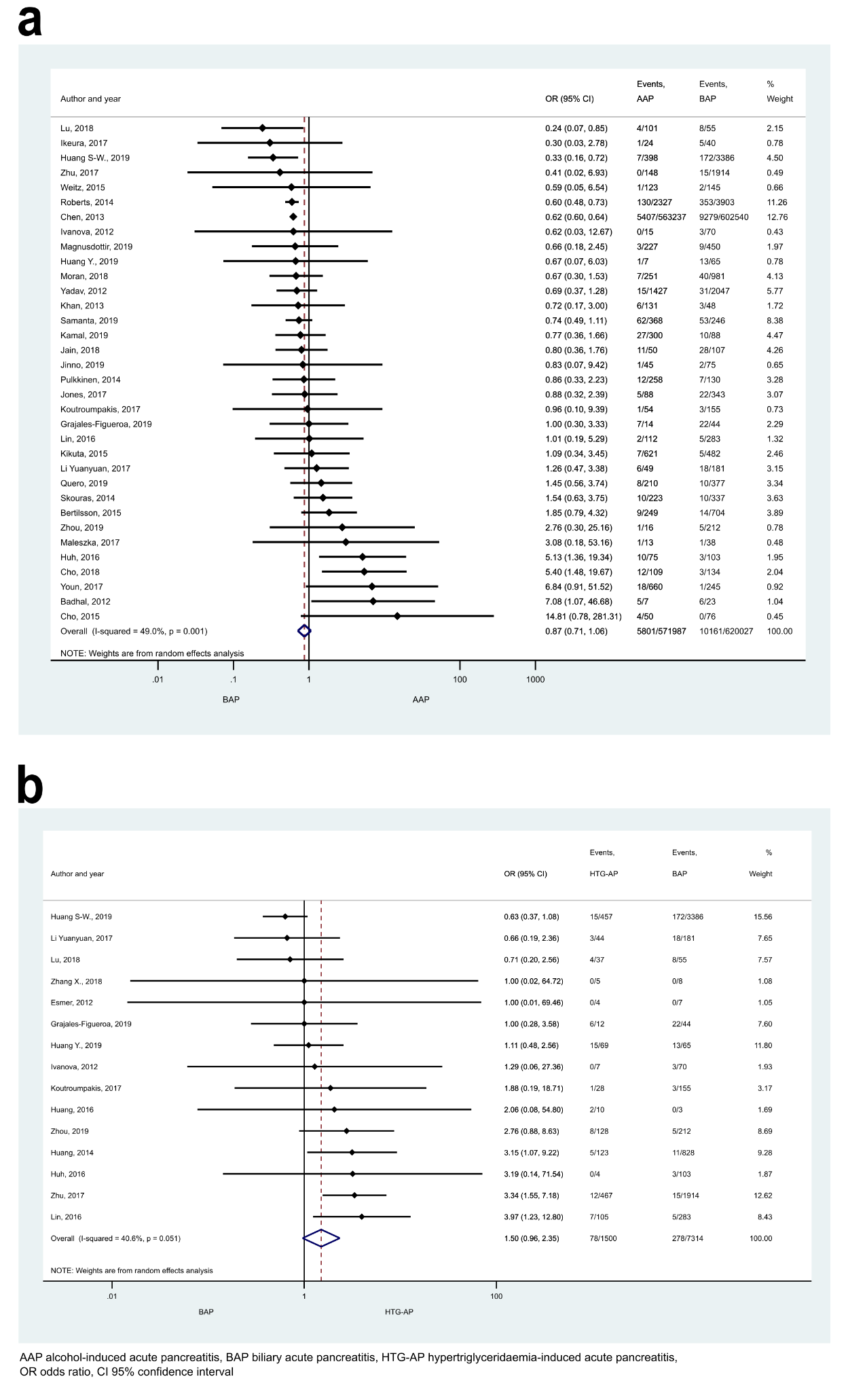


**Supplementary Figure S14.** Forest plot showing the effect of different disease aetiologies on mortality. The effects of (a) AAP and PAP, p=0.673; (b) BAP and PAP, p=0.987. Filled rhombuses represent the ORs derived from the articles analysed. Horizontal bars represent CI. Empty rhombus shows the overall OR (the middle of the rhombus, CIs are the edges).


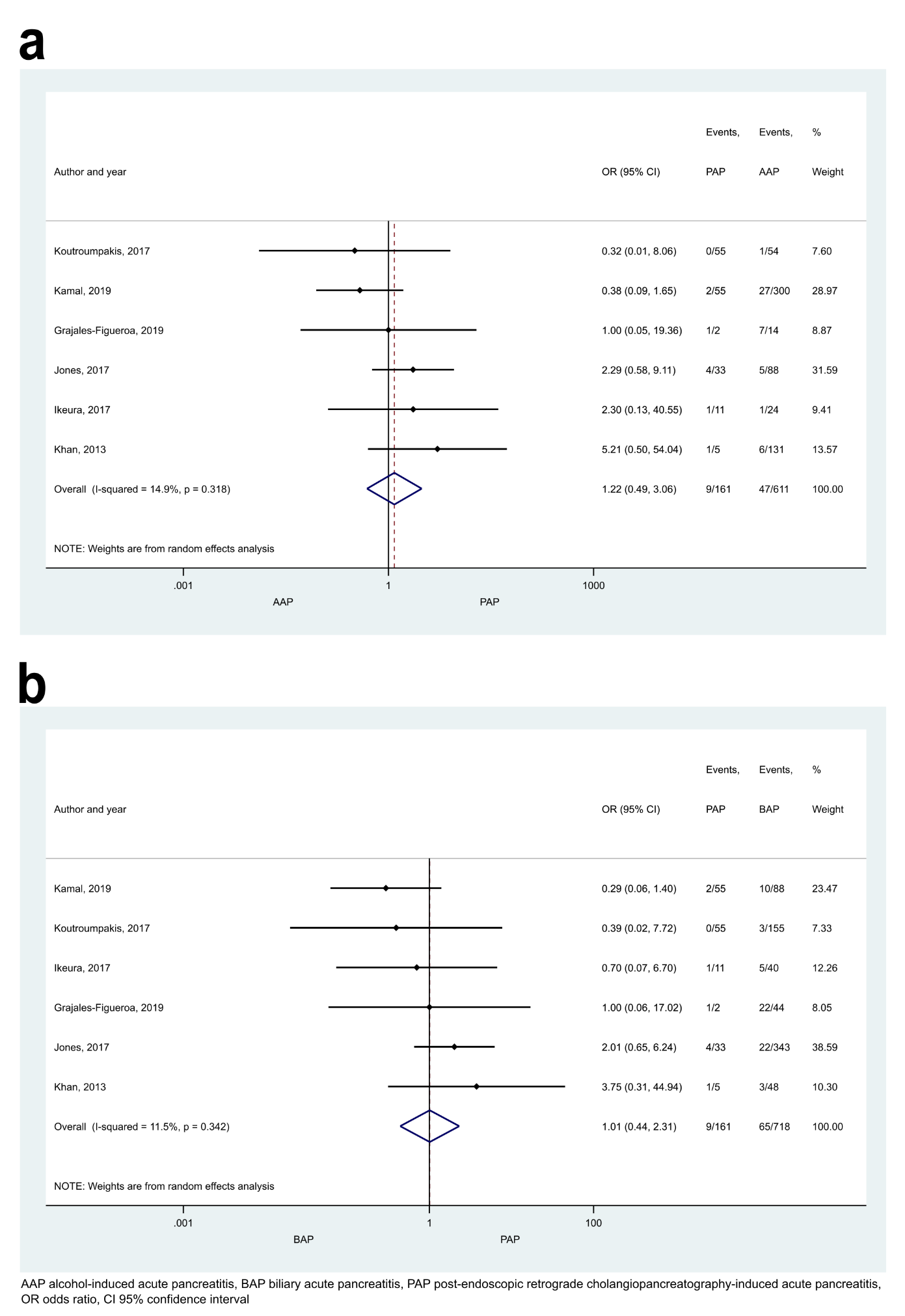


**Supplementary Figure S15.** Sensitivity analysis related to the Forest plot of mortality, AAP vs. BAP (Supplementary Figure S13a).


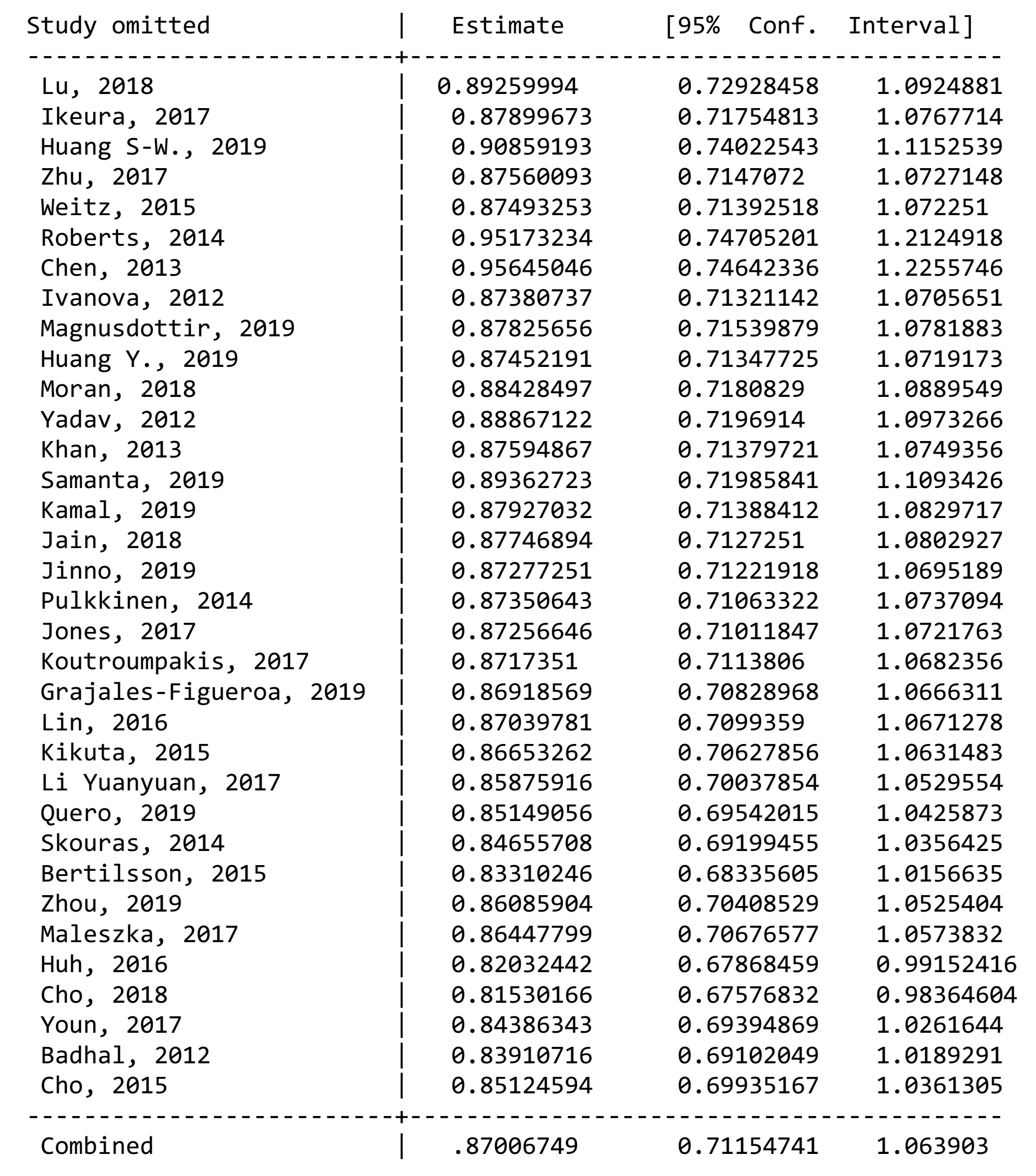


**Supplementary Figure S16.** Funnel plots of mortality related to Figure 6, Supplementary Figure S13. (a) AAP vs. HTG-AP, p=0.865; (b) BAP vs. HTG-AP, p=0.328; (c) AAP vs. BAP, p<0.001. The two oblique lines mark the pseudo-95% confidence limits.


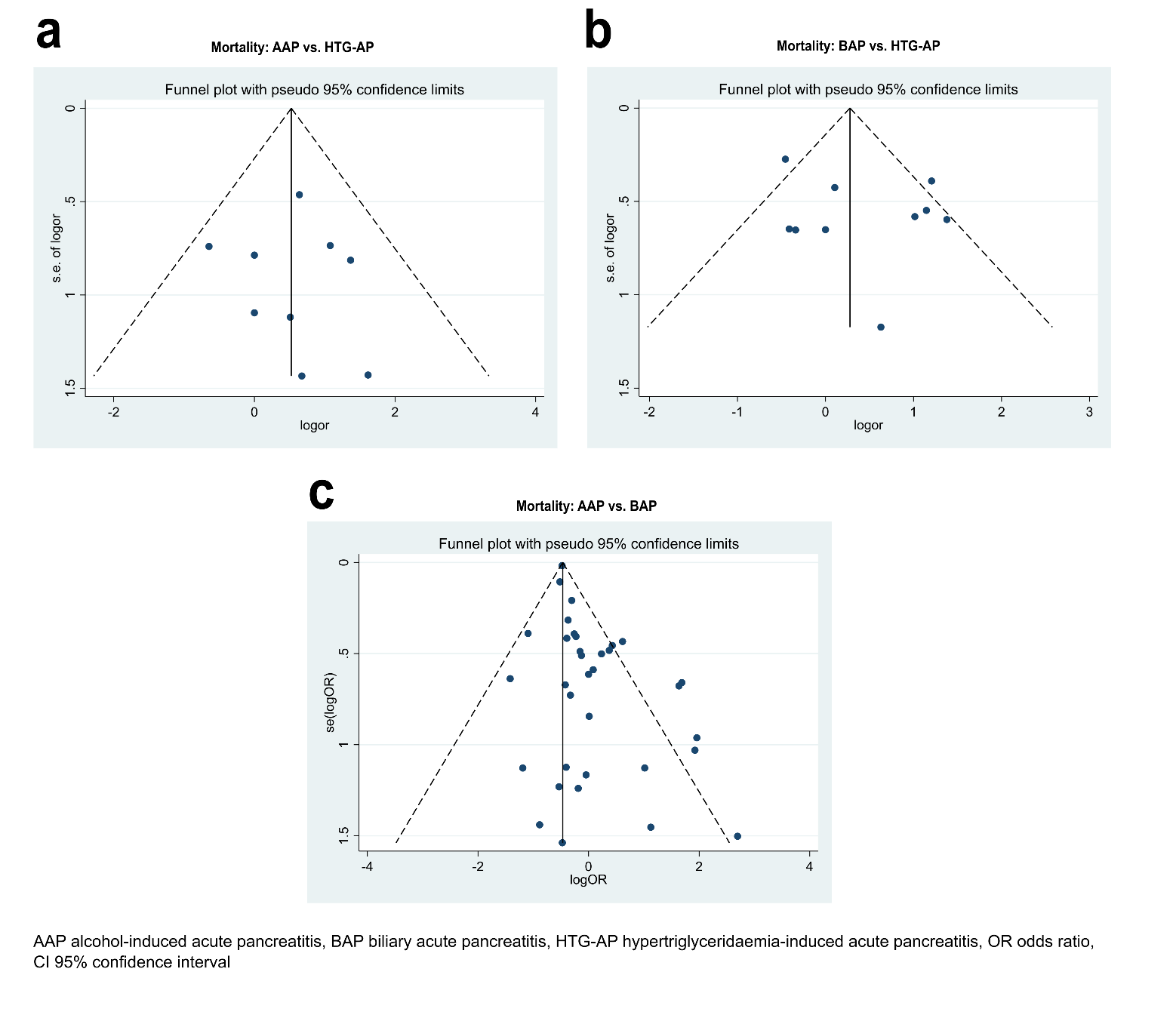


**Supplementary Figure S17. Pseudocyst, fluid collection** Forest plot showing the effect of different disease aetiologies on fluid collection and pseudocyst. The effects of (a) AAP and BAP on fluid collection, p=0.228; (b) HTG-AP and BAP on pseudocyst, p=0.183; (c) AAP and BAP on pseudocyst, p=0.259. Filled rhombuses represent the ORs derived from the articles analysed. Horizontal bars represent CI. Empty rhombus shows the overall OR (the middle of the rhombus, CIs are the edges).


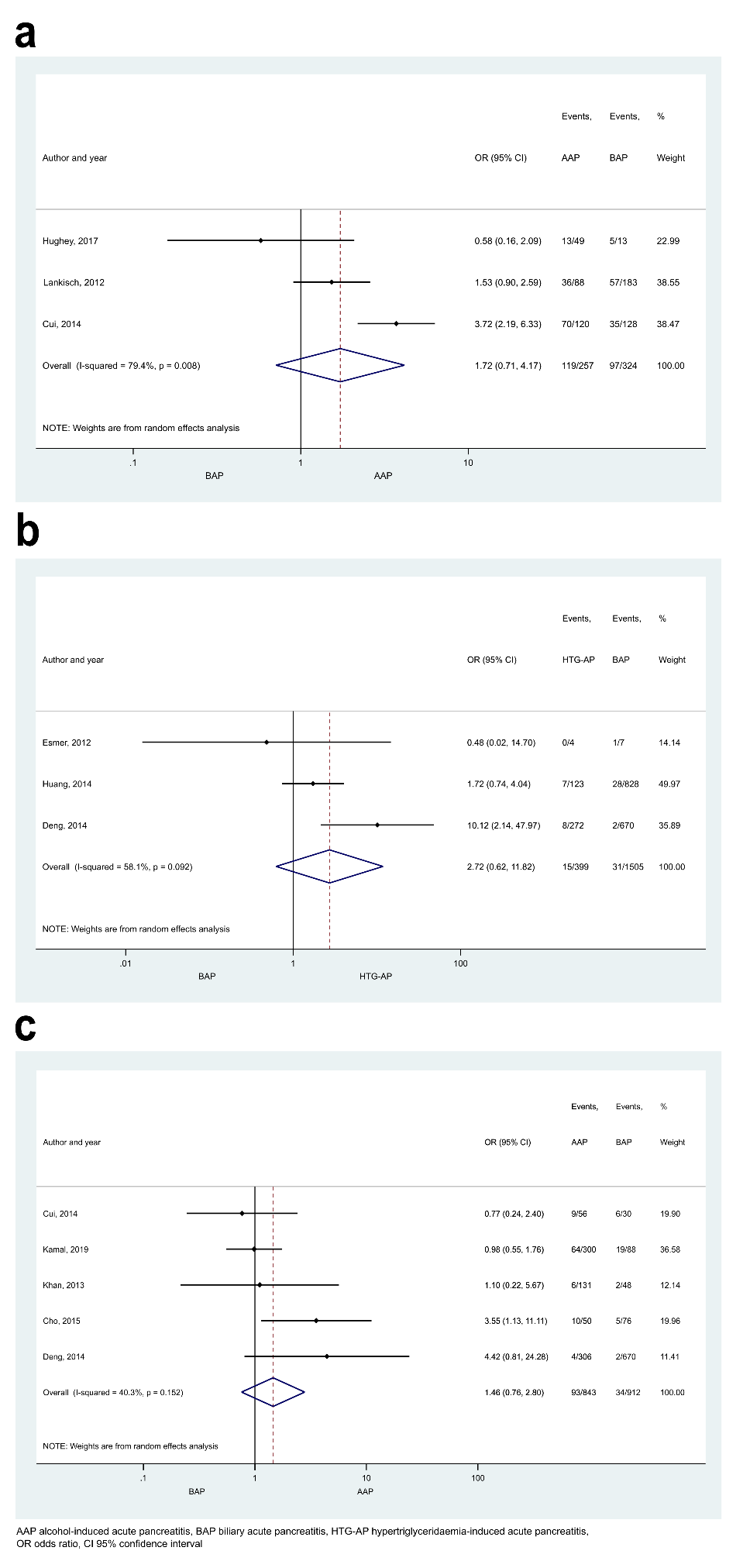

Supplement: Supplementary file 1 — Supplementary Information [file 41598_2020_74943_MOESM1_ESM.docx]
